# Quasi-Solid Electrolyte Interphase Boosting Charge and Mass Transfer for Dendrite-Free Zinc Battery

**DOI:** 10.1007/s40820-023-01031-7

**Published:** 2023-02-28

**Authors:** Xueer Xu, Yifei Xu, Jingtong Zhang, Yu Zhong, Zhongxu Li, Huayu Qiu, Hao Bin Wu, Jie Wang, Xiuli Wang, Changdong Gu, Jiangping Tu

**Affiliations:** 1 State Key Laboratory of Silicon Materials, Key Laboratory of Advanced Materials and Applications for Batteries of Zhejiang Province, and School of Materials Science and Engineering, Zhejiang University, Hangzhou, 310027 People’s Republic of China; 2 Institute for Composites Science Innovation (InCSI) and State Key Laboratory of Silicon Materials, School of Materials Science and Engineering, Zhejiang University, Hangzhou, 310027 People’s Republic of China; 3 Qingdao Industrial Energy Storage Research Institute, Qingdao Institute of Bioenergy and Bioprocess Technology, Chinese Academy of Sciences, Qingdao, 266101 People’s Republic of China; 4 Zhejiang Laboratory, Hangzhou, 311100 People’s Republic of China; 5 Department of Engineering Mechanics, School of Aeronautics and Astronautics, Zhejiang University, Hangzhou, 310027 People’s Republic of China

**Keywords:** Mass transfer, Defect engineering, Quasi-solid electrolyte interphase, Zinc metal anode, Zinc batteries

## Abstract

The practical applications of zinc metal batteries are plagued by the dendritic propagation of its metal anodes due to the limited transfer rate of charge and mass at the electrode/electrolyte interphase. To enhance the reversibility of Zn metal, a quasi-solid interphase composed by defective metal–organic framework (MOF) nanoparticles (D-UiO-66) and two kinds of zinc salts electrolytes is fabricated on the Zn surface served as a zinc ions reservoir. Particularly, anions in the aqueous electrolytes could be spontaneously anchored onto the Lewis acidic sites in defective MOF channels. With the synergistic effect between the MOF channels and the anchored anions, Zn^2+^ transport is prompted significantly. Simultaneously, such quasi-solid interphase boost charge and mass transfer of Zn^2+^, leading to a high zinc transference number, good ionic conductivity, and high Zn^2+^ concentration near the anode, which mitigates Zn dendrite growth obviously. Encouragingly, unprecedented average coulombic efficiency of 99.8% is achieved in the Zn||Cu cell with the proposed quasi-solid interphase. The cycling performance of D-UiO-66@Zn||MnO_2_ (~ 92.9% capacity retention after 2000 cycles) and D-UiO-66@Zn||NH_4_V_4_O_10_ (~ 84.0% capacity retention after 800 cycles) prove the feasibility of the quasi-solid interphase.

**Supplementary Information:**

The online version contains supplementary material available at 10.1007/s40820-023-01031-7.

## Highlights

- Defect engineering for constructing Zn^2+^ reservoir to anchor anions.
- The quasi-solid electrolyte interphase as Zn^2+^ reservoir boosting charge and mass transfer for dendrite-free zinc battery.
- A Coulombic efficiency of 99.8% was achieved in Zn||Cu cell.

**Supplementary Information:**

The online version contains supplementary material available at 10.1007/s40820-023-01031-7.

## Graphical abstract

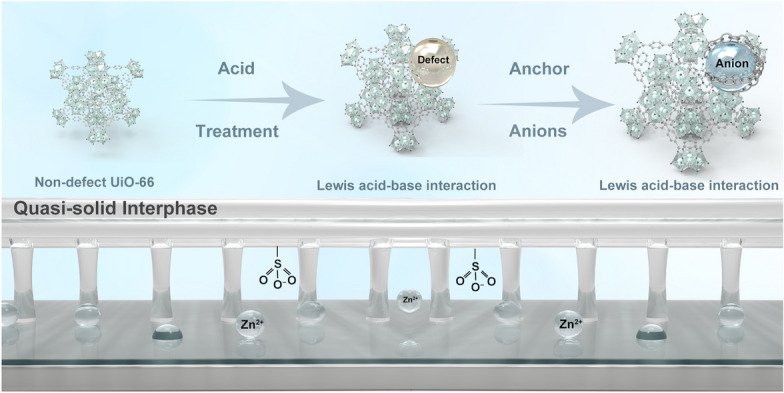

## Introduction

Zinc-ion batteries (ZIBs) have provided a promising complement to lithium-ion batteries for electrochemical energy storage [1–3]. To date, metallic Zn is recognized as an ideal anode material for ZIBs in terms of its high gravimetric capacity (820 mAh g^−1^), moderate redox potential (− 0.76 V vs .standard hydrogen electrode), abundant resource, and nontoxicity [4–8]. However, uncontrollable dendrites formed on the anode result in irreversible capacity loss, further hindering the practical application of zinc metal batteries (ZMBs) [9].

Tremendous efforts have been performed to enhance the durability and reversibility of Zn anodes, including modifying the Zn electrode structure [10], building artificial protective layers [11–14], and introducing electrolyte additives [15–17]. Among them, metal–organic frameworks (MOFs) have emerged as one of the most promising materials to mitigate the Zn dendrite growth [18, 19]. However, MOFs in previous literatures are usually utilized as ionic sieves for their porous structures. The ZMBs with these ionic sieve interphases are still susceptible to severe polarization and dendrite-related failure after repeated cycles especially when both high current densities and capacities are applied [20]. Fundamentally, controlling the migration process of the charge carrier is still challenging because transport-limited dendritic zinc growth still exists for the reported artificial interfaces. In specific, dendrite growth is intrinsically related to both charge and mass transfer during the electrochemical process. Due to the limited diffusivity of cations, the high current density accelerates zinc ion depletion at the electrode/electrolyte interphase, creating a severe electrolyte concentration gradient in the vicinity of the anode. Such a concentration gradient increases the ionic flux near the protruded sites, rendering the protrusions to grow into dendrites. Additionally, uneven distribution of the Zn^2+^ ion flux also leads to the formation of inhomogeneous Zn crystals which would further evolve into the dendrites by a root-growth behavior. As the Zn dendrites are prone to be detached from the substrate, ZMBs suffer from the severe decay of the reversible capacity and the Coulombic efficiency (CE). More seriously, the accumulation of dead Zn could easily pierce the separator, leading to battery failure [21, 22].

The sand’s capacity (*C*_sand_) is a critical parameter to elucidate the dendrite formation mechanism [23]. It can be determined via the following equation:

$$C_{{\text{Sand}}} = \pi D_{\text{app}}\frac{{(z_{\text{c}}c_{\text{0}}F)^{2} }}{{4J(1 - t_{\text{c}})^{2} }}$$

in which, *Z*_*c*_ and *t*_*c*_ represent the Zn^2+^ charge number and transference number, respectively. *c*_*0*_ is the salt concentration, *D*_app_ is the diffusion coefficient and *F* is the Faraday’s constant. According to the Sand’s formula, below sand’s capacity, dense root-growing Zn deposits are developed in the battery due to the reaction-controlled process. Thereby, the preferable deposition of Zn could be realized by enhancing the safety limit. In addition to reduced local current densities, guided by this theoretical model, increasing the salt concentration, ionic conductivity (which is related to the *D*_app_) or zinc–ion transference number are all effective strategies in solving Zn dendrite issues.

Inspired by this model, we herein report a novel quasi-solid interphase on the anode surface to inhibit the dendrite growth by comprehensively maximizing the sand’s capacity. The artificial layer is composed of the rich content of defective UiO-66 (D-UiO-66) nanoparticles, one of the promising metal–organic frameworks (MOFs), and extremely low content of electrolytes inside the MOF channels. The positively charged oxygen vacancies of the D-UiO-66 would immobilize anions of the electrolyte via Lewis acid–base interaction. This fixation not only impedes the migration of anions but also forms anion-decorated MOF channels to facilitate the transport of Zn^2+^, increasing the cation transference number ultimately. Additionally, the interchannel liquid phase endows the layer with high ionic conductivity. Noteworthy, the infiltrated electrolyte inside the MOF scaffolds is highly concentrated because of the partial desolvation within the porous layer. Such layer structure acts as a zinc ions reservoir, mitigating the concentration polarization and enabling a homogeneous Zn-ion distribution. Therefore, Zn anodes with the quasi-solid interphase layers achieve superior electrochemical Zn plating/stripping performance for more than 1800 h at 1 mAh cm^−2^ (average Coulombic efficiency of 99.8%), even extending a long cycling lifetime of over 480 h at 5 mAh cm^−2^. Moreover, Zn||MnO_2_ full cell delivers prominent cycling stability with a capacity retention of 92.9% after cycling for 2000 cycles, while Zn||NH_4_V_4_O_10_ full cell also possesses an impressive capacity retention of ~ 84% after 800 cycles at 8 A g^−1^.

## Experiment Section

### Synthesis of Non-Defect UiO-66

The ND-UiO-66 was prepared according to a reported hydrothermal approach [24]. ZrCl_4_ (3.728 g) and H_2_BDC (5.316 g) were dissolved in 96 mL of DMF and concentrated HCl (2.86 mL) was then added at room temperature under continuous stirring for 1 h. The above mixture was transferred into a Teflon lined autoclave and heated to 220 °C for 16 h. After naturally cooling down to room temperature, the ND-UiO-66 powders were collected by centrifugation, successively washed with DMF three times and dried under vacuum at 160 °C.

### Synthesis of Defective UiO-66

The defective UiO-66 was prepared according to a reported hydrothermal approach. ZrCl_4_ (0.96 g), H_2_BDC (5.016 g) and different equivalents (5, 10, and 20) of HBC with respect to H_2_BDC were dissolved in 240 mL of DMF. After continuous stirring for 2 h, the above mixture was transferred into a Teflon lined autoclave and heated to 120 °C for 22 h. After naturally cooling down to room temperature, the white precipitate was centrifuged, washed by DMF three times and dried under vacuum at 60 ℃ overnight. The resulting product was immersed in dilute HCl solution to make the defects. The sample was dried at 120 °C overnight and further heated at 350 °C (under vacuum) for 12 h to thermally activate the D-UiO-66.

### Preparation of UiO-66 Coated Zn Anode

The defective UiO-66 (or defect-free UiO-66) was dispersed into DME by stirring and sonicating. The homogeneous dispersion was dropped on Zn foils and dried in vacuum at 80 ℃ for 5 min. Then the Zn foils continuously passed through the rotating rollers to make the structure compact.

## Result and Discussion

### Materials Synthesis and Characterization

**Fig. 1:**
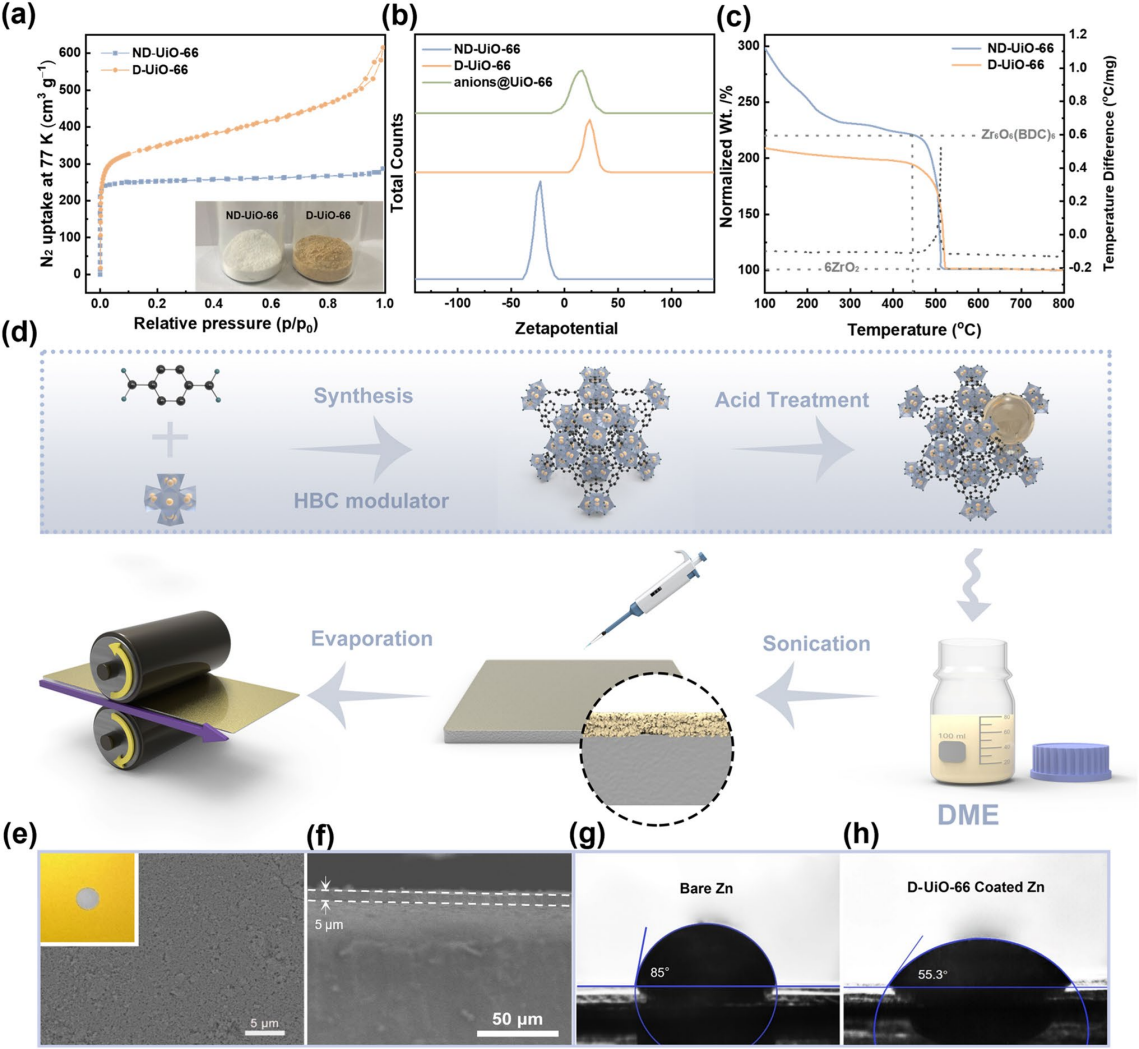
**a** Nitrogen adsorption–desorption isotherms at 77 K of ND-UiO-66 and D-UiO-66. The inset shows the color change between the two UiO-66. **b** Zeta potential curves of UiO-66 in different statuses. **c** TGA–DSC curves of UiO-66. Solid curves, left axis, TGA trace (relative to ZrO_2_). Dotted curves, right axis, DSC trace. **d** Illustration depicting the synthetic route of the D-UiO-66 layer. **e** Top-view and **f** cross-sectional SEM images of D-UiO-66-based layer. The inset of **e** shows the optical image of D-UiO-66@Zn. Images of contact angles between electrolyte and **g** D-UiO-66@Zn **h** or bare Zn

MOFs are rather a new class of highly crystalline and nanoporous materials. Among them, UiO-66 is a prototypical Zr-MOF constructed from Zr_6_ building clusters and 1,4 benzenedicarboxylate (BDC) linkers, which is selected in consideration of its stability in water [25]. After being modulated by benzoic acid (HBC) and attacked by the acid treatment, UiO-66 frameworks generate missing-linker defects, leading to unsaturated Zr centers (i.e., open metal sites, OMSs) [26]. X-ray powder diffraction (XRD) pattern of defective UiO-66 (D-UiO-66) is in accordance with the non-defect UiO-66 (ND-UiO-66) and the simulated pattern (Fig. S1), indicating that the crystal structure of the D-UiO-66 remains intact. To validate the presence of defects in the frameworks, N_2_ adsorption–desorption isotherms (Fig. 1a) were measured at 77 K. The Brunauer–Emmett–Teller (BET) surface area of D-UiO-66 is 1470 m^2^ g^–1^, which is larger than that of the ideal one (1251 m^2^ g^–1^). In addition, the color of UiO-66 powders changes from white to light yellow (inset of Fig. 1a) with the generation of defects, owing to the ligand-to-metal charge transfer [27]. Moreover, compared with the ND-UiO-66, the removal of organic linkers in the framework results in positive-charged UiO-66 powers (D-UiO-66), as verified by *zeta* potential (Fig. 1b). Such positively charged OMSs promote preferential coordination with anions. As expected in anions@UiO-66, *zeta* potential shifts to negative potential after anchoring the anions.

The thermogravimetric analysis-differential scanning calorimetry (TGA–DSC) traces were used to quantitatively calculate the number of missing-linker defects. The calculation method was proposed by the previous report [28]. As shown in Fig. 1c, the significant weight loss in the 450–530 ℃ range is accompanied with an intense exothermic heat peak at around 500 °C in the DSC curve (dashed grey curve), which originates from the decomposition of organic linkers. Missing linker defects are commonly studied according to the chemical equation:

$${\text{Zr}}_{6}{\text{O}}_6({\text{BDC}})_{6} ({\text{s}}) + 45 {\text{O}}_{2} ({\text{g}}) \to 6{\text{ZrO}}_2 + 48{\text{CO}}_{2}\;({\text{g}}) + 12{\text{H}}_{2}{\text{O (g)}}$$

The molar mass of ideal UiO-66 (Zr_6_O_6_(BDC)_6_) is 2.2 times as heavy as 6 mol of ZrO_2_, which is assumed as the only solid residue at 800 °C. Thus, if the end weight of ZrO_2_ is normalized to 100%, after the loss of solvent, the dehydroxylated UiO-66 (Zr_6_O_6_(BDC)_6_) should be just found at 220% on the TGA trace. In fact, it could be observed that the plateau of ND-UiO-66 reaches 220% before the decomposition of ligands at 445 °C, in turn emphasizing the sample ideality. Differently, the weight of D-UiO-66 is lower than the ND-UiO-66 at the same temperature, indicating that the linker deficiencies have originally existed in the framework. According to the weight loss, the number of missing linkers can be calculated (see detailed assignments in Table S1). Noticed that the defect concentration in the UiO-66 increases with the increase of HBC content in the synthesis (Fig. S2). Compared with the ND-UiO-66 whose metal clusters are 12-coordinated, when adding ten equivalents of HBC with respect to H_2_BDC, the coordination number of Zr_6_ octahedron is 10.72, which is represented the D-UiO-66 in this work.

An artificial MOF film was elaborately prepared on the Zn metal anode by a solvent evaporation reaction, as illustrated in Fig. 1d. Specifically, the D-UiO-66 (or ND-UiO-66) was dispersed into DME by stirring and sonicating. Then the homogeneous dispersion was dropped on the Zn foil and dried in vacuum at 80 °C for 5 min. After that, the coated foil continuously passed through the rotating rollers to make an intimate contact between the Zn metal and the MOF layer. Benefited from this strategy, the interfacial stability to withstand volume changes of Zn anode during the Zn plating/stripping process is enhanced. Moreover, UiO-66 nanoparticles might chemically interact with the Zn surface via dangling carboxyl groups, which also ensure a tight contact [29]. As shown in the digital photo (inset of Fig. 1e), such a MOF layer is dense and uniform. Even under microscopic observation with the scanning electron microscopy (SEM), it is still extremely homogeneous (Fig. 1e). To demonstrate the hydrophilic interface, the contact angle (CA) was measured by dropping the liquid electrolyte on the bare Zn and UiO-66 coated Zn (Fig. 1g, h). MOF-coated Zn (55.3°) exhibits a significantly lower wetting angle than that of bare Zn (85°), suggesting excellent electrolyte absorption capability of the UiO-66 coating layer. The hydrophilic interface allows the liquid electrolyte to impregnate the porous MOF nanocrystals adequately.

### Mechanism Investigation

After contacting with the electrolyte, the MOF layer encapsulates the electrolyte solution in the pores, forming the quasi-solid artificial interphase layer. To further confirm the specific characteristics of this unique layer, we compare the D-UiO-66 with and without pretreatment. In specific, the pretreated UiO-66 (denoted as E@UiO-66) was first soaked with the electrolyte solution and then collected after filtration and removal of any excessive water [30]. BET and Fourier transform infrared spectroscopy (FTIR) were performed to probe the successful impregnation of the electrolyte inside the channels of the MOF. In comparison with the aforementioned defective UiO-66 (1470 m^2^ g^–1^), the BET surface area (Fig. S3) of the E@D-UiO-66 dramatically decreases to near 150 m^2^ g^–1^. The pore size distribution of D-UiO-66 are presented in Fig. S4b [31, 32]. Moreover, from the FTIR analysis (Fig. S4), most characteristic peaks of both UiO-66 and E@D-UiO-66 are consistent with the reported value of UiO-66 [33]. Specifically, the sharp peaks appeared at 1350 and 1395 cm^−1^ are attributed to asymmetric stretching and symmetric stretching vibrations of carboxylic functional groups (–COO–) in the terephthalic acid [33, 34], while the minor absorption band at 1506 cm^−1^ is caused by the typical vibrations of carbon–carbon double bond (C=C) in the benzene ring [35]. However, new peaks were clearly detected in the E@D-UiO-66. The strong characteristic peaks at 983, 1054, and 1130 cm^−1^ represent symmetric stretching and asymmetric stretching vibrations of SO_4_^2−^ [36], whereas others appearing at 588 cm^−1^ (deformation vibration) and 1234 cm^−1^ (asymmetric stretching vibration) are associated with the SO_3_ group in the E@D-UiO-66 [37].

**Fig. 2:**
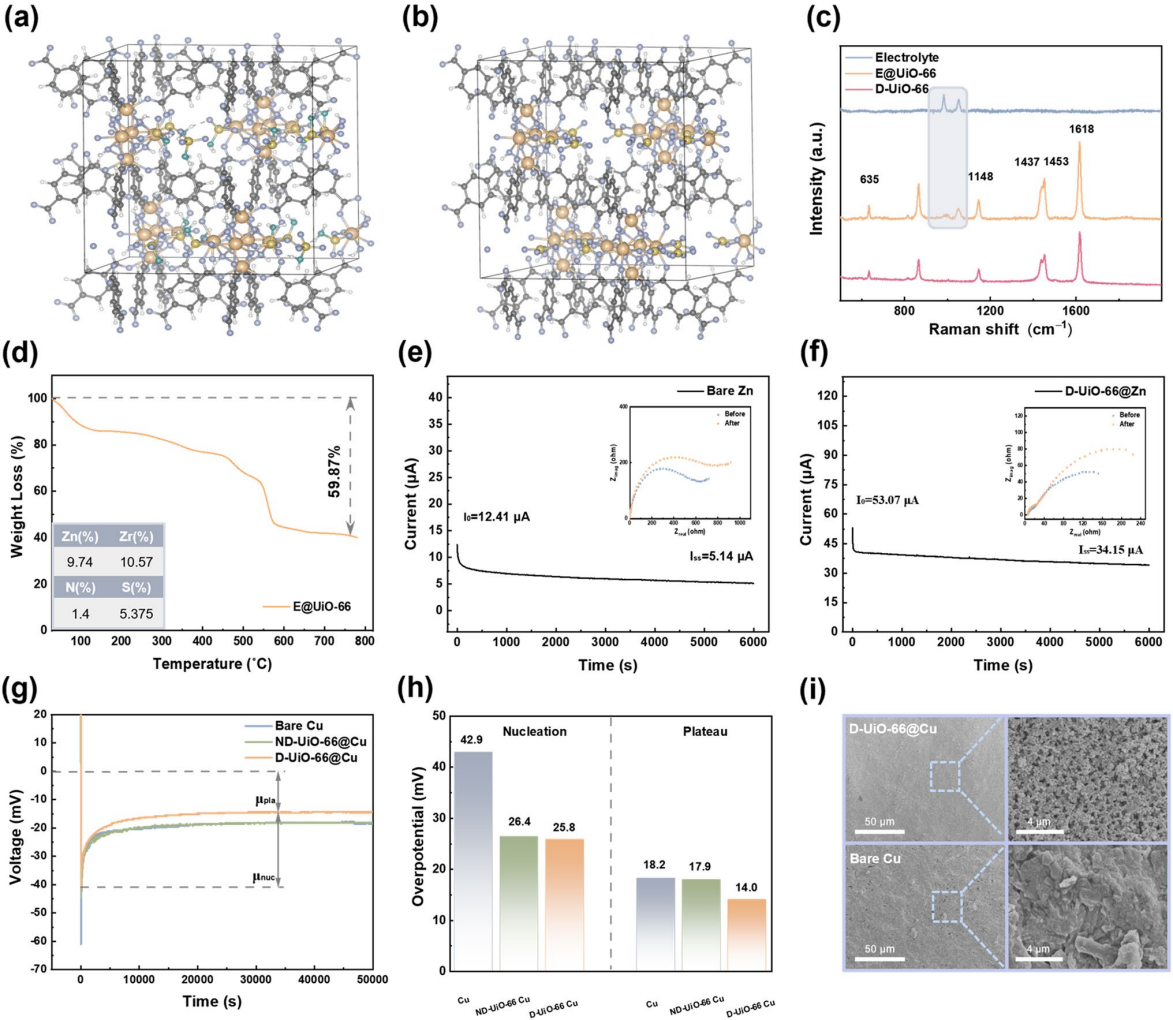
DFT calculations of **a** NH_2_SO_3_^−^ and** b** SO_4_^2−^ adsorbed on the defect sites (white, gray, yellow, purple, orange, and cyan represent hydrogen, carbon, sulfur, oxygen, zirconium, and nitrogen atoms, respectively). **c** Raman spectra of E@D-UiO-66 in comparison with liquid electrolyte and D-UiO-66 nanoparticles without pretreatment. **d** TGA curve of E@D-UiO-66 in air. Inset shows the result of ICP-AES. Current–time profile for **e** Zn||Zn symmetric cell and **f** D-UiO-66@Zn||D-UiO-66@Zn symmetric cell with potentiostatic polarization (*∇V* = 10 mV). Insets show AC impedance spectra before and after the following polarization. **g** Voltage profiles of Zn deposition on various Cu substrates at 0.5 mA cm^−2^ and the** h** corresponding overpotential in the different regions. **i** SEM images of Zn deposition morphology with and without quasi-solid artificial interphase

In the presence of the electrolyte (1 M ZnSO_4_ and 1 M Zn(NH_2_SO_3_)_2_), anions spontaneously bind to the unsaturated coordinative Zr sites forming anion-decorated channels of D-UiO-66 [38]. Density functional theory (DFT) calculation (Fig. 2a, b) and Raman spectroscopy (Figs. 2c and S5) provide additional insights into the complexation of anions with OMS via Lewis acid–base interaction [39]. According to the theoretical results, the binding energy between NH_2_SO_3_^−^ anions and the defect sites is − 4.85 eV, while that between SO_4_^2−^ and the same sites is − 10.26 eV. In parallel, from Raman spectra, both UiO-66 and E@UiO-66 exhibit the featured peaks associated with the BDC ligands at 635, 1437, 1148, 1453, and 1618 cm^−1^ (see detailed analysis in Table S2) [40], in line with the previous report. Concomitantly, E@D-UiO-66 shows emerging peaks at 994 and 1049 cm^−1^, which is ascribed to the SO_4_^2−^ and SO_3_ group, respectively. Nevertheless, compared with the liquid electrolyte, the absorption band corresponds to the SO_3_ group shifts from 1052 to 1049 cm^−1^ (Fig. S5a), which experimentally validates the coordination of NH_2_SO_3_^−^ to the OMS [37]. Introducing the functional group –SO_3_ was reported to homogenize the zinc deposition [41]. On the other hand, the peak assigned to the SO_4_^2−^ shifts from 981 cm^−1^ towards a higher frequency region with the electrolyte concentration increasing (Fig. S5b). In the UiO-66 pore, the *ν*SO_4_^2–^ vibration clearly shifts towards higher frequency (ca. 994 cm^−1^), indicating that the highly concentrated electrolyte is achieved inside UiO-66 channels [42]. Furthermore, the formula of E@UiO-66 was determined using coupled plasma atomic emission spectroscopy (ICP-AES), Elemental analysis (EA), and thermogravimetric analysis (TGA) (Fig. 2d). Zn/Zr molar ratio was obtained by ICP-AES of 0.92, N/S molar ratio was examined by EA of 0.62 and the content of solvent within E@UiO-66 was revealed by TGA, which verified a nominal formula of E@D-UiO-66 as Zr_6_O_4_(OH)_4_(BDC)_4.72_[Zn(NH_2_SO_3_)_2_]_3.49_(ZnSO_4_)_4.189_(H_2_O)_20.4_ (see details in Supporting Information). The low H_2_O/Zn molar ratio (≈2.65) suggests that each Zn^2+^ is solvated by less than three water molecules in the confining electrolyte within the MOF comparable to that of solvated [Zn(H_2_O)_6_]^2+^ species in the bulk electrolyte. Apart from the aforementioned Raman spectra, the composition provides further convincing evidence for high concentration Zn^2+^ (evaluated as ≈21 M, Supporting Information) within the D-UiO-66, which is significant thanks to the partial desolvation process of the layer.

To examine the ion-transport behavior within the channels of UiO-66 nanoparticles intrinsically, zinc transference number (*tZn*^*2*+^) and ionic conductivity (*σ*) were measured. The transference number of ND-UiO-66@Zn electrodes was calculated to be 0.37 (Fig. S6), which is slightly higher than that of the bare Zn electrodes (0.20) (Fig. 2e). However, when introducing the defects in UiO-66, the *tZn*^*2*+^ is impressively as high as 0.60 (Fig. 2f), which again proves the fixation of anions to the positively charged oxygen vacancies. Meanwhile, instead of sacrificing the conductivity to increase the *t*Zn^2+^, the D-UiO-66 coating layer possesses excellent ionic conductivity (evaluated as ≈6.90 × 10^−3^ S cm^−1^, Fig. S7). The unique ion-transport properties are possibly ascribed to three reasons: (i) anion-decorated MOF channels (especially negative charged –SO_3_) could facilitate the Zn^2+^ migration but delay the transport of water molecules; (ii) liquid phase in the pore channels of UiO-66 ensures the fast Zn^2+^ diffusion kinetics; (iii) high concentrated electrolyte within the pores confers quasi-solid interphase with the function of zinc ions replenishment.

### Zn Plating/Striping Behavior with Quasi-Solid Artificial Interphase

Due to the promotion of Zn^2+^ transport in the quasi-solid interphase, we tentatively hypothesized that Zn deposition–dissolution behaviors could be efficiently modulated. Accordingly, the symmetric cells and the asymmetric cells (using Zn as counter/reference electrode) were assembled to investigate this hypothesis. It is found that the cyclic voltammetry (CV) curves (Fig. S8a–e) of the D-UiO-66 coated cell exhibit a more intense and reversible current response than the other two cells, implying faster charge transfer kinetics and superior reversibility induced by modified layer. As depicted in Fig. 2g, h, galvanostatic (at a fixed current density of 0.5 mA cm^−2^) voltage profiles display the nucleation overpotential (*μ*_nuc_) and the plateau potential (*μ*_pla_), which is controlled by the mass-transfer. Nucleation overpotential refers to voltage that drives the nucleation of Zn embryos, while plateau overpotential is associated with the growth of zinc after initial nucleation [43]. In comparison with the bare Cu and ND-UiO-66@Cu, a notably decreased nucleation overpotential is achieved in D-UiO-66@Cu, as low as 25.8 mV (versus bare Cu of 42.9 mV, ND-UiO-66@Cu of 26.4 mV). Similarly, the plateau potential (14.0 mV) is also slightly lower than the other counterparts. The above results verify that the D-UiO-66 layer lowers the energy barrier of Zn nucleation and growth, due to its capability to promote the rapid Zn^2+^ migration and tailor the solvated Zn^2+^ clusters. Therefore, D-UiO-66@Cu displays a flat and intact coating (Fig. 2i) without any obvious protrusion after 50 cycles. By contrast, the whole desolvation process occurs on the bare electrode surface (Fig. S9). Porous and rough structure induced by heavy zincate accumulation could be observed on the bare Cu (Fig. 2i).

**Fig. 3:**
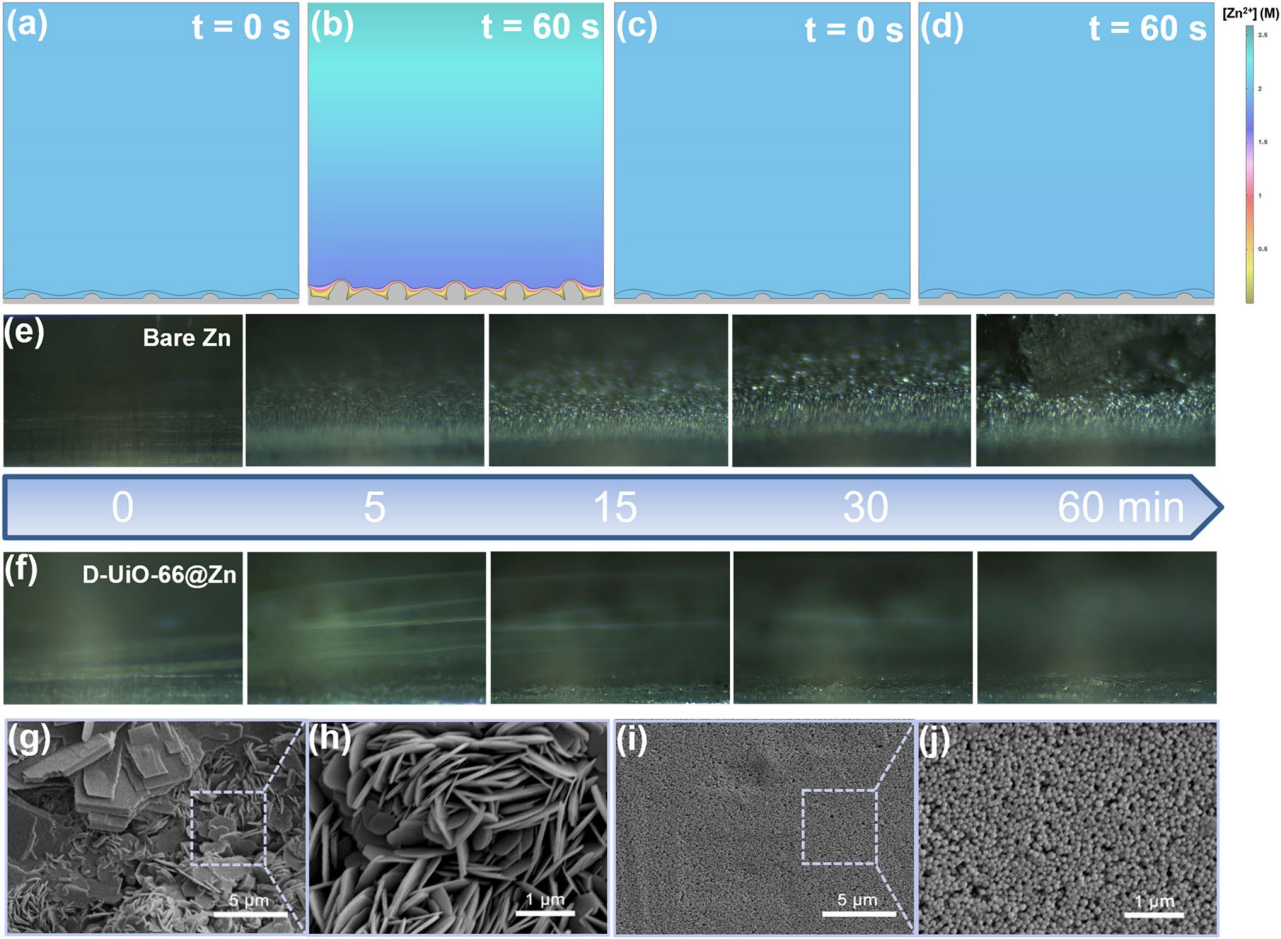
**a–d** Simulated zinc ions concentration profiles and Zn deposition of **a, b** the bare Zn anode and **c, d** D-UiO-66@Zn anode before and after 60 s. The Zn deposition process of **e** bare Zn and **f** D-UiO-66@Zn using an *in-situ* optical microscope at a current density of 5 mA cm^−2^. SEM images for **g, h** bare Zn anode and **i, j** D-UiO-66@Zn anode after 50 plating/stripping cycles

The continuous deposition behavior is highly related to the concentration field distribution at the anode/electrolyte interface. Finite element modeling (FEM) was employed herein to simulate the concentration distribution near the Zn surface in the Zn deposition process (Fig. 3a–d). Since bare Zn contacts with electrolyte directly, the passivation layer (Zn_4_SO_4_(OH)_6_ · *x*H_2_O) would be formed inevitably, which is also imitated on the bare Zn surface in this simulation [44]. As shown in Fig. 3b, a rapid consumption of zinc ions near the passivation layer creates a Zn^2+^ deficiency, resulting in a severe concentration gradient. In comparison, the fixation of anions and continuous liquid transport environment endow the quasi-solid protective layer with both high *t*Zn^2+^ and *σ*Zn^2+^. Thanks to these features, the Zn^2+^ concentration distribution is uniform and the concentration of Zn^2+^ near the quasi-solid interface is higher than that near the passivation layer of the bare Zn (Fig. 3d). Notably, the highly concentrated electrolyte inside the MOF channel is not considered in the simulation. Such enrichment of ions remarkably replenishes the Zn^2+^ consumption, further reducing the concentration gradient near the Zn metal surface. The concentration gradient leads to concentration polarization in electrolytes, which is regarded as the key reason for dendrite growth. In terms of efficacy, the D-UiO-66 layer could serve as a dendrite inhibitor. During the zinc plating process, homogeneous Zn^2+^ concentration distribution suppresses the dendrite propagation. Comparatively, the large Zn–salt concentration gradient near the passivation layer accelerates the propagation of Zn dendrite, promoting nuclei growth into pillar-like Zn granules.

To visually examine the depression of Zn dendrite formation by the quasi-solid interface, *in-situ* optical microscope was adopted. The optical images (Fig. 3e, f) show the surface morphology evolution of Zn electrodes (bare Zn and D-UiO-66@Zn) during the deposition process with a high current density of 5 mA cm^−2^. The bare Zn electrode exhibits a smooth surface at the initial time. However, after 15 min, visible protrusions unevenly grow on the bare Zn anode and gradually turn into dendritic Zn in the following time. In the contrast, no portent of serious agglomeration or Zn dendrite is observed in the D-UiO-66 coated Zn all the time. SEM was further conducted to observe the morphology of the Zn deposition upon cycling at micro-scale. After several Zn platting/stripping cycles, bare Zn presents obvious morphology variation including Zn flakes as well as the regular hexagon with sharp tips (Fig. 3g, h), while D-UiO-66@Zn still remains dense and intact (Fig. 3i, j). Note that the deposition morphologies are known to be diverse at different current densities [45]. The morphology differences evidence that the unique quasi-solid layer structure could regulate the distribution of the electric field. That attributes to the three-dimensional porous matrix with a large surface area, which could guide a homogenous ion flux and limits local current density to some extent [46]. Coupled with the aforementioned reduction of the concentration polarization, the dendrite formation through a root-growth mechanism can be effectively avoided [47–49].

**Fig. 4:**
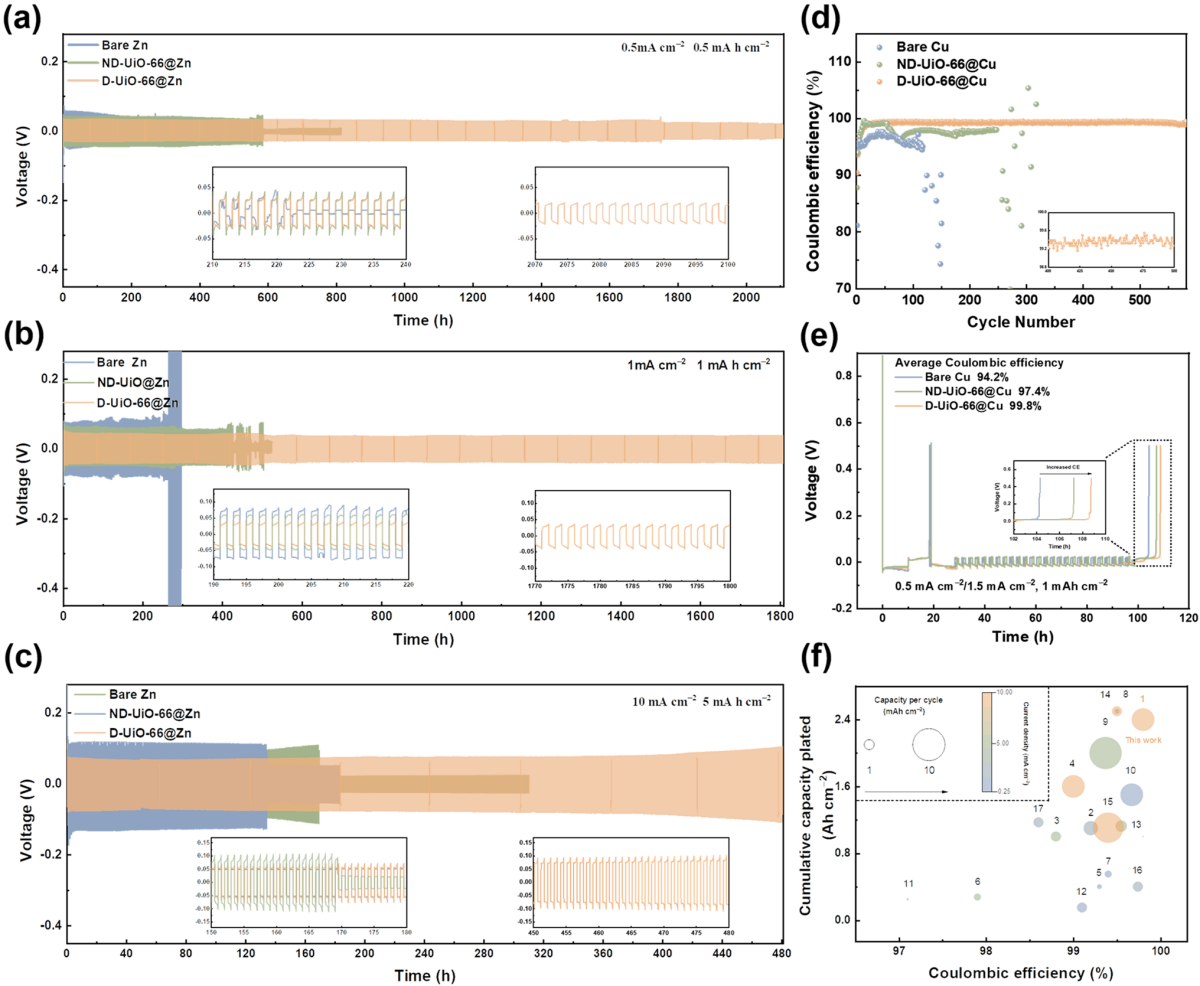
Electrochemical Performance of half cells. Galvanostatic Zn plating/stripping of various zinc anodes in a symmetric cell at **a** 0.5 mA cm^−2^ and 0.5 mAh cm^−2^, **b** 1 mA cm^−2^ and 1 mAh cm^−2^, and **c** 10 mA cm^−2^ and 5 mAh cm^−2^. **d** Coulombic efficiency (CE) of Zn plating/striping on various electrodes at 1 mA cm^−2^ and 0.5 mAh cm^−2^.** e** Voltage–time curves to measure the average CE of Zn plating/striping on various electrodes. **f** Comparison of per-cycle areal capacity, plating current density, CPC, and the CEs in recent reports. The detailed references corresponding to the point number and other related reports are listed in Table S3, supporting information

The efficacy of the quasi-solid interphase toward enhancing the long-term stability of the Zn anode was gauged by galvanostatic cycling of symmetric Zn half cells. By measuring the Zn plating/stripping stability of different linker deficiencies (Fig. S10), it can be observed that the optimized number of defects is when the amount of HBC is ten equivalents with respect to H_2_BDC (denoted as D-UiO-66). As depicted in Fig. 4a, at 0.5 mA cm^−2^ and 0.5 mAh cm^−2^, the D-UiO-66@Zn electrode delivers an ultralong lifespan of 2100 h, which is much longer than that of the bare Zn electrode (≈220 h) and ND-UiO-66@Zn (≈580 h). Similarly, at 1 mA cm^−2^ and 1 mAh cm^−2^, the D-UiO-66@Zn electrode could run for over 1800 h at a marginally lower voltage hysteresis of 66 mV (Fig. 4b). In contrast, the sudden failure appears after a few limited cycles for bare Zn and ND-UiO-66@Zn, which may be due to the dendrite accumulation. This cycling trend is analogous to that under more aggressive conditions (10 mA cm^−2^, 5 mAh cm^−2^). The D-UiO-66@Zn based symmetrical cell exhibits a superior durability and obviously lower voltage hysteresis for more than 480 h, while short circuits occur in the other two cells after several cycles (Fig. 4c). The excellent cycling performance is due to the ability of the quasi-solid interface to eliminate concentration polarization and homogenize the ion flux, fundamentally expanding the sand’s capacity and thus suppressing the dendrite growth.

The Coulombic efficiency (CE) is a critically important parameter used to evaluate the reversibility of the Zn anode [50, 51], which is determined by the Zn||Cu asymmetrical cells at a current of 1 mA cm^−2^ and a capacity of 0.5 mAh cm^−2^ (Figs. 4d and S11). The CE of bare Cu and ND-UiO-66@Cu exhibit fluctuant voltage signals and rapidly decrease within 100 cycles and 300 cycles, respectively, whereas a relatively higher and stable coulombic efficiency of 99.4% is remained for more than 550 cycles with the existence of D-UiO-66 layer. Furthermore, owing to the ameliorative interfacial compatibility, the overpotential of D-UiO-66@Cu anode reduces by nearly 47.2% from 70.0 mV at the initial state to 33.1 mV at 500 cycles (Fig. S11e, f). A standard “zinc reservoir” protocol was further adopted to accurately quantify this value (see details in the Supporting Information) [20]. D-UiO-66 coated electrode delivers an average CE of 99.8% (Fig. 4e), outperforming the counterparts of bare Cu (94.2%) and ND-UiO-66@Cu (97.4%). These differences are ascribed to the homogeneous ion flux and rapid Zn^2+^ transport induced by the quasi-solid interphase. To accurately assess the modified systems in recent reports, an effective comparison was drawn in terms of four key test parameters: per-cycle areal capacity, plating current density, cumulative plated capacity (CPC), and the CEs. Among the published efforts (Fig. 4f and Table S3), the D-UiO-66 layer shows an impressive CPC (2400 mAh cm^−2^) and CE (≈99.8%) at 10 mA cm^−2^ and 5 mAh cm^−2^, suggesting its effects on the protection of the Zn anode in ZMBs.

### Electrochemical Performance of Full Cells

**Fig. 5:**
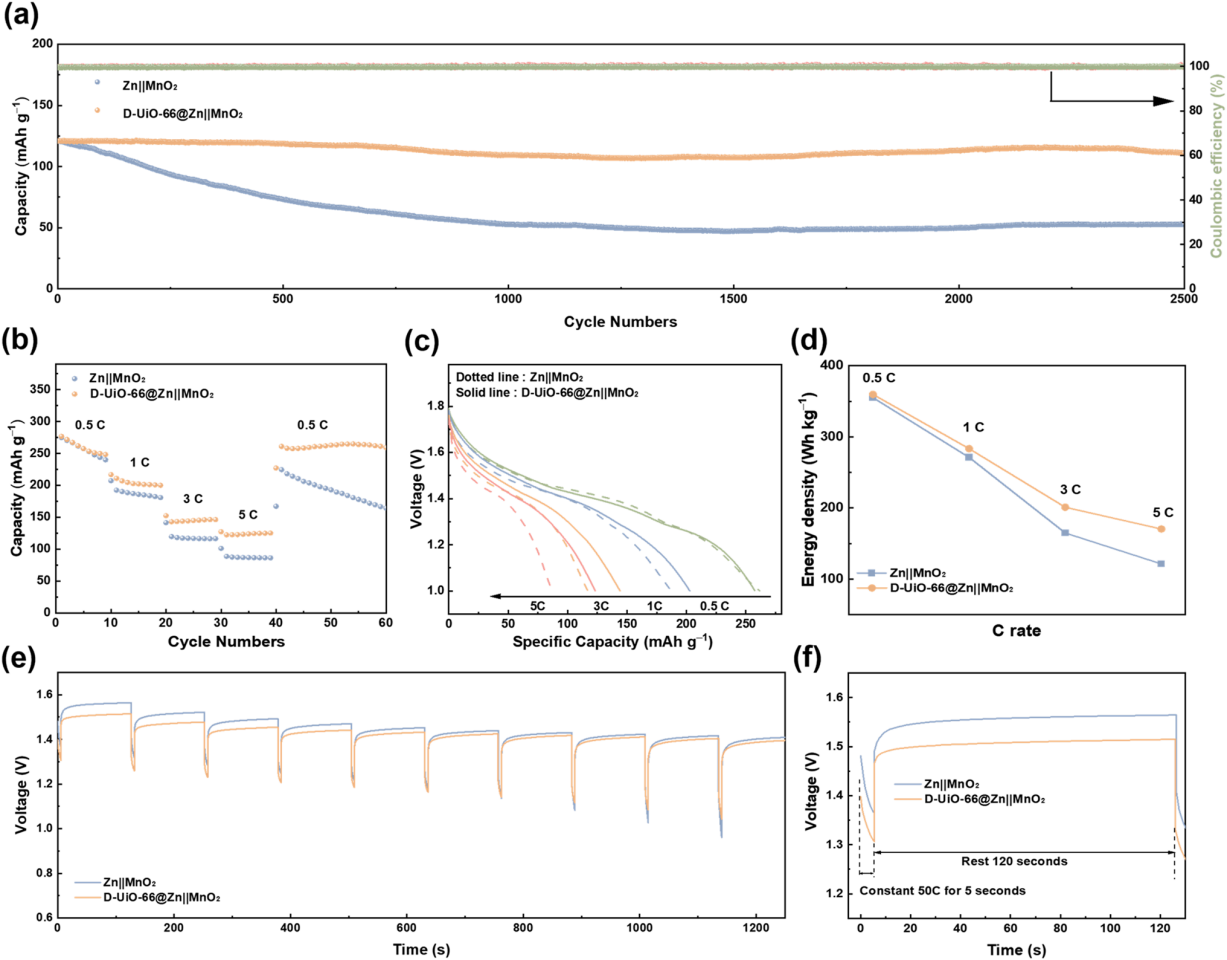
Performances of Zn||MnO_2_ full cells. **a** Long-cycling performance, **b** rate performance and **c** corresponding discharge profiles of Zn||MnO_2_ full cells with and without D-UiO-66-based quasi-solid interphase. **d** Energy output at different C-rate. **e** voltage–time profiles of the pulse discharge test at a C-rate of 50 and **f** the magnified view at the first 120 s

The feasibility in the practical application of the quasi-solid interphase in rechargeable AZIBs was demonstrated in full cell chemistry comprising the *δ*-MnO_2_ (XRD result in Fig. S12) cathodes coupled with Zn anodes [52, 53]. Besides, we use 0.2 M MnSO_4_ as the electrolyte additive to inhibit the dissolution of Mn^2+^ from the cathodes [54]. Both two CV curves deliver similar shapes of typical and noticeable redox peaks (Fig. S13), consistent with the reverse conversion reactions between Mn^3+^ and Mn^4+^. Galvanostatic charge–discharge tests (Fig. 5a) were conducted on Zn||MnO_2_ cells at 25 °C in a voltage range of 1.0–1.8 V at 5C (1C corresponding to 380 mA g^−1^). Using the D-UiO-66-coated Zn anode, a robust capacity of ≈111.0 mAh g^−1^ could be obtained after 2500 cycles with a ≈100% CE under the same condition, exhibiting a high-capacity retention of ~ 92.5%. Whereas for the bare Zn anode, the capacity of the full cell declines rapidly, where only ~ 52.7 mAh g^−1^ specific capacity is retained under the same condition, corresponding to the 43.3% reversible capacity retention. Zn dendrites growth derived from the large polarization could be responsible for this inferior Zn reversibility. Moreover, D-UiO-66@Zn||MnO_2_ battery manifests an excellent rate performance at various C rates from 0.5C to 5C (Fig. 5b, c). The modulated battery well outperforms the reference battery with the C rates increasing, even though they possess almost similar initial capacity (276.8 mAh g^−1^). When the C rates return back to 0.5C, the capacity of D-UiO-66@Zn||MnO_2_ battery can recover to 261.0 mAh g^−1^. Figure 5d further compares the energy output of the batteries at different C rates, where D-UiO-66@Zn||MnO_2_ battery shows superior energy density than the Zn||MnO_2_ battery, especially at higher C rates. At a C rate of 5, the modulated and reference cells deliver an energy output of 170 and 121 Wh kg^−1^ (based on the mass of MnO_2_), respectively. The excellent energy output mainly stems from the reduced concentration polarization. To further quantitatively examine the effect of quasi-solid interphase on mitigating the concentration polarization, a pulse discharging protocol (discharging: 50C for 5 s; resting:120 s) was applied for 10 cycles (Fig. 5e, f) [55]. High-rate discharging accelerates concentration polarization and the following resting period dissipates this phenomenon. As expected, D-UiO-66@Zn||MnO_2_ battery is 28.2% faster (18.36 vs.25.56 s) to reach 99% of the final battery voltage than that of the bare Zn||MnO_2_ battery, manifesting the effectiveness of quasi-solid interphase in alleviating concentration polarization.

**Fig. 6:**
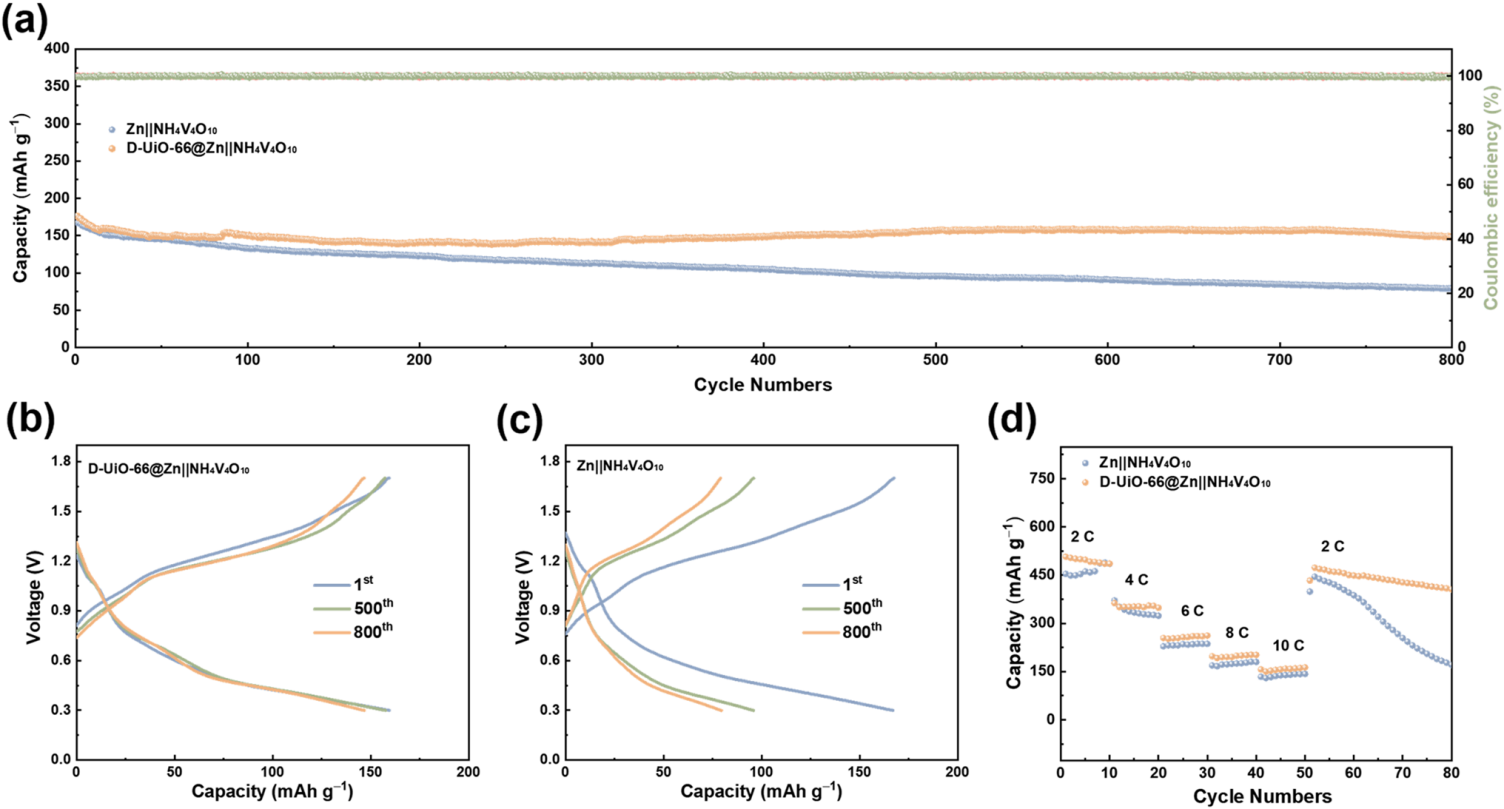
Performance of Zn||NH_4_V_4_O_10_ full cells. **a** Long-cycling performance and corresponding discharge profiles of Zn||NH_4_V_4_O_10_ full cells **b** with and **c** without D-UiO-66-based quasi-solid interphase. **d** Rate performance of Zn||NH_4_V_4_O_10_ full cells with and without D-UiO-66-based quasi-solid interphase

The versatility of quasi-solid interphase could be further validated in full cells consisting of a promising cathode NH_4_V_4_O_10_ (XRD result in Fig. S14) coupled with D-UiO-66@Zn anode. Both Zn||NH_4_V_4_O_10_ and D-UiO-66@Zn||NH_4_V_4_O_10_ cells display similar behaviors with distinct V-ion redox peaks (Fig. S15), aligning with the previous work [56]. In the case of the galvanostatic cycling performance at 8 A g^−1^ (based on the NH_4_V_4_O_10_ mass), D-UiO-66@Zn||NH_4_V_4_O_10_ maintains a capacity retention of ~ 84.0% for over 800 cycles. On the contrary, the reversible discharge capacity of the Zn||NH_4_V_4_O_10_ battery degrades drastically by 47.5% after 800 cycles (Fig. 6a). The corresponding charge–discharge curves also attest that more reversible and steady battery operation is achieved with the protection of the D-UiO-66 layer (Fig. 6b, c). Moreover, D-UiO-66@Zn||NH_4_V_4_O_10_ cell also exhibits superior rate performance with the range of C rate from 2 to 8C (Fig. 6d). Especially when the current density is reduced to 2C, D-UiO-66@Zn||NH_4_V_4_O_10_ cell also delivers excellent cycling stability, while the bare Zn||NH_4_V_4_O_10_ cell suffers from continuous capacity decay. Therefore, the novel interfacial design in this literature offers an effective strategy to achieve high-performance aqueous ZMBs.

## Conclusions

In summary, a quasi-solid electrode/electrolyte interphase is formed on Zn anode by introducing a defective UiO-66 layer, within which the liquid electrolyte is filled. The quasi-solid electrolyte layer serves as a zinc ions reservoir. In specific, the defective UiO-66 layer possesses Lewis acidic sites to bind with anions, reducing the anionic mobility. Combining the liquid phase with the functionalization by anions, the MOF channels accelerate cations transportation inside and thus improves the *σ*Zn^2+^ and* t*Zn^2+^, further promoting the mass transfer significantly. Moreover, the nanoporous structure not only partially rejects water molecules to form a high concentrated solution in the channels but also lowers the local current density. Hence, guided by the sand’s capacity model, such a versatile interfacial layer could suppress the dendrite growth via expanding the safety limit of ZMBs. The symmetrical batteries exhibit long-term cycling stability and ultra-stable overpotential for more than 2000 h at 0.5 mA cm^−2^ and 0.5 mAh cm^−2^. Even at high current densities and high deposition capacities (10 mA cm^−2^, 5 mAh cm^−2^), the cell still maintains an endurable plating/stripping performance for more than 480 h. Furthermore, D-UiO-66@Zn||MnO_2_ battery delivers a high-capacity retention of ~ 92.5% at 5C after 2500 cycles, while D-UiO-66@Zn||NH_4_V_4_O_10_ achieves the cyclability of ~ 84.0% capacity retention at 8 A g^−1^ for over 800 cycles. This strategy can also promisingly prevent dendrite growth in Mg–metal batteries as well as other metal batteries facing the same issues, taking a step towards their use in energy-storage applications.

### Supplementary Information

Below is the link to the electronic supplementary material.

Supplementary file1 (PDF 1260 KB)
