# Supplementary material for: Quasi-Solid Electrolyte Interphase Boosting Charge and Mass Transfer for Dendrite-Free Zinc Battery
Source: Nanomicro Lett. 2023 Feb 28;15:56. doi: 10.1007/s40820-023-01031-7 (PMC9975136; doi:10.1007/s40820-023-01031-7)
Supplement: Supplementary file 1 — Supplementary file1 (PDF 1260 KB) [file 40820_2023_1031_MOESM1_ESM.pdf]

Supporting Information for

Quasi-Solid Electrolyte Interphase Boosting Charge and Mass Transfer for Dendrite-Free Zinc Battery

Xueer Xu[1], Yifei Xu[2], Jingtong Zhang[4], Yu Zhong[1], ZhongXu Li[1], Huayu Qiu[3], Hao Bin Wu[2], Jie Wang[4, 5], Xiuli Wang[1], Changdong Gu[1, *], Jiangping Tu[1]

[1] State Key Laboratory of Silicon Materials, Key Laboratory of Advanced Materials and Applications for Batteries of Zhejiang Province, and Department of Materials Science and Engineering, Zhejiang University, Hangzhou 310027, P. R. China

[2] Institute for Composites Science Innovation (InCSI) and State Key Laboratory of Silicon Materials, School of Materials Science and Engineering, Zhejiang University, Hangzhou 310027, P. R. China

[3] Qingdao Industrial Energy Storage Research Institute, Qingdao Institute of Bioenergy and Bioprocess Technology, Chinese Academy of Sciences, Qingdao, 266101, P. R. China

[4] Zhejiang Laboratory, Hangzhou 311100, Zhejiang, P. R. China

[5] Department of Engineering Mechanics, School of Aeronautics and Astronautics, Zhejiang University, Hangzhou 310027, P. R. China

* Corresponding author. E-mail: cdgu@zju.edu.cn (Changdong Gu)

S1 Supplemental Experimental Procedures

S1.1 Fabrication of MnO_2 Cathode

The CC@MnO_2 was fabricated by a facile hydrothermal process. In detail, the commercial carbon cloth was immersed into an 80 mL Teflon-lined stainless-steel autoclave with 50 mL of 0.01 M $KMnO_4$ aqueous solution, followed by heating at 160 °C for 1 h. After that, the product was cleaned by deionized water and dried at 60 °C.

S1.2 Fabrication of $NH_4V_4O_{10}$ Cathode

The $NH_4V_4O_{10}$ cathode was synthesized by the classic hydrothermal method. 5 mmol ammonium metavanadate was dissolved in 80 °C deionized water. Subsequently, 2 mmol of oxalic acid and 1 mmol of ammonium fluoride were added to the solution. Then, the obtained solution was transferred into Teflon-lined stainless-steel autoclave and treated by the hydrothermal condition of 140 °C for 48 h. The reaction product was centrifuged and dried to obtain $NH_4V_4O_{10}$ powder. Working electrodes were prepared using a slurry coating technology by blending $NH_4V_4O_{10}$, Super-P, and polyvinylidene fluoride (PVDF) in a weight ratio of 7:2:1 with N-methyl-2-S3 pyrrolidone (NMP)

solvent. The uniform slurry was then coated onto a Ti foil and then vacuum-dried at 80 °C for 10 h. The loading mass of $NH_4V_4O_{10}$ in the electrode was ~1.5 mg cm^{-2}.

S1.3 Assembly of Symmetric Cells and Full Cells

CR2025-type coin symmetric cells were assembled with identical electrodes of bare Zn or UiO-66@Zn, 1 M $ZnSO_4$ and 1 M $Zn(NH_2SO_3)_2$ electrolyte and glass fiber separators. Zn||MnO_2 cells were assembled by using bare Zn or UiO-66@Zn as anodes, 1 M $ZnSO_4$|1 M $Zn(NH_2SO_3)_2$|0.2 M $MnSO_4$ as the electrolyte, and glass fiber as separators. Zn||$NH_4V_4O_{10}$ cells were assembled by using bare Zn or UiO-66@Zn as anodes, 1 M $ZnSO_4$|1 M $Zn(NH_2SO_3)_2$ as the electrolyte, and glass fiber as separators. All batteries were assembled in open air conditions and aged for 4 hours before performed electrochemical measurements.

S1.4 Material Characterizations

Powder X-ray diffraction (PXRD) patterns were recorded on an RIGAKU D/MAX 2550/PC instrument equipped with a copper Kα radiation (k = 1.54 Å) in a 2θ range from 5° to 55°. The water contact angle was measured at room temperature using a contact angle meter (SL200B, Solon Tech.). Raman spectra were determined on a Renishaw inVia Raman microscope with a 785 nm laser. Fourier transform infrared (FTIR) spectroscopy analysis was carried out using 100-FT-IR Spectrometer, Perkin-Elmer, with a wavenumber resolution of 4 cm^{-1} to characterize UiO-66. The zeta potential was measured using a Malvern Zetasizer Nano ZS90. Nitrogen adsorption–desorption measurements at 77 K were performed on an Autosorb-iQ2-MP (Quantachrome Instruments) surface area analyzer. Prior to the measurement, the sample was outgassed under vacuum at 443 K for 12 h. Thermal gravimetric analysis (TGA) profiles and Thermal gravimetric analysis-differential scanning calorimetry (TGA-DSC) profiles were obtained on a SDT Q600 V8.2 Build 100 system under a mixture of nitrogen and oxygen from 308 K to 1073 K with a heating rate of 5 K min^{-1}. Zirconium and zinc ratio was collected by inductively coupled plasma optical emission spectrometer (ICP-OES, Agilent 5110). Sulfur and Nitrogen ratio was obtained by elemental analyzer (elementarvarioel cube). The morphologies were characterized using a field-emission scanning electron microscope (SEM, Hitachi S-4800) with an accelerating voltage of 3.0 kV and the energy dispersive spectroscopy (EDS). To prepare solid-like electrolyte@UiO-66, activated UiO-66 were fully soaked in 1 M $ZnSO_4$|1 M $Zn(NH_2SO_3)_2$ and collected by vacuum filtration. The *in-situ* optical observation of Zn deposition/dissolution behavior was carried out by pairing symmetrical Zn (or UiO-66@Zn) in a cell with a transparent quartz window (Kejing, STC-Q) and a current density of 10 mA cm^{-2}.

The ionic conductivity of the quasi-solid interphase was measured using two blocking electrodes (stainless steel). The ionic resistance R_b was tested by the EIS technique. Then the thickness of the quasi-solid interphase layer L, and the contact area S were measured. The ionic conductivity of the E@UiO-66 was evaluated according to the following equation: $\sigma = \frac{L}{R_b S}$

Based on the result of ICP-AES, the formula of E@D-UiO-66 is determined as $Zr_6O_4(OH)_4(BDC)_{4.72}[Zn(NH_2SO_3)_2]_{3.49}(ZnSO4)_{4.189}(H2O)_{20.4}$. In the TGA measurement, the initial two-step weight loss (in total of ~ 11.51%) up to 100°C is attributed to the decomposition of apparent H_2O. The subsequent weight drop originates from the disintegration of MOF structure (leaves Zirconium Oxide), decomposition of zinc salts and water molecules in the pore channels. Inductively coupInductively coupled plasma mass-spectrometry (ICP-MS) analysis of E@D-UiO-66 confirmed a Zn:Zr ratio of 1:1.09. Elemental analysis (EA) of E@D-UiO-66 confirmed a N:S ratio of 1:3.8. The remaining weight (40.13%) corresponds to a mixture of ZrO_2 and ZnO, and the value of x can be deduced from the following equation: 40.13% / (10.57 × $M(ZrO_2)$ + 9.74 × M(ZnO)) = 100% / $M(Zr_6O_4(OH)_4(BDC)_{4.72}[Zn(NH_2SO_3)_2]_{3.49}(ZnSO_4)_{4.189}(H_2O)_x)$, where $M(ZrO_2)$, M(ZnO), and $M(Zr_6O_4(OH)_4(BDC)_{4.72}[Zn(NH_2SO_3)_2]_{3.49}(ZnSO4)_{4.189}(H_2O)_x)$ are the molecular weights of ZrO_2, ZnO and the E@D-UiO-66, respectively. Based on the calculated molecular weight of E@D-UiO-66, the nominal formula is determined as $Zr_6O_4(OH)_4(BDC)_{4.72}[Zn(NH_2SO_3)_2]_{3.49}(ZnSO4)_{4.189}(H_2O)_{20.4}$. Thus, the concentration of Zn^{2+} was calculated as around 21 M.

S1.5 Electrochemical Measurements

Cyclic voltammetry (CV) curves and Linear Sweep Voltammetry (LSV) were conducted on an electrochemical workstation (CHI 660D) with a voltage window of 1.0 to 1.8 V at different scan rates. The galvanostatic charge/discharge (GCD) measurements were performed by a battery test system (LAND, CT-2001A). Electrochemical impedance spectra (EIS) were measured in a frequency range from 100 kHz to 1 mHz. Zn||Cu (or UiO-66@Cu) half cells were assembled to evaluate the Coulombic efficiency (CE) of Zn plating/stripping.

Method 1: A fixed amount of Zn was deposited on UiO-66@Cu or bare Cu foil, followed by stripping Zn until the voltage reached up to 0.6 V.

Method 2: In order to carry out an accurate CE, a method proposed by Wang et al. was adopted. According to the protocol, a fixed Zn capacity of 4 mA h cm^{-2} was deposited onto the Cu substrate as a “zinc reservoir” and then cell repeated the plating/stripping process for 25 cycles with 1 mA h cm^{-2}, followed by stripping away the remaining Zn to calculate the average CE.

The transference number of Zn^{2+} ($t_{Zn^{2+}}$) was determined by the EIS technique and the potentiostatic polarization. Symmetrical bare Zn (or ND-UiO-66@Zn, D-UiO-66@Zn) battery cells were polarized by a constant voltage bias of 10 mV (ΔV) for 90min. The initial current (I_0) and the steady-state current (I_{SS}) were recorded in combination with the interfacial resistance before (R_0) and after (R_{SS}) the potentiostatic polarization. The $t_{Zn^{2+}}$ thereby could be calculated by the following equation:

$$t_{Zn^{2+}} = \frac{I_{ss}(\Delta V / I_0 R_0)}{I_0(\Delta V / I_{ss} R_{ss})}$$

S1.6 Computational Section

Our DFT calculations are performed with Vienna Ab initio Simulation Package (VASP) in this work [1], and the PBEsol with D3 dispersion correction is used as the electron exchange-correlation potential [2, 3]. The cutoff energy of plane wave basis is 500 eV. The structure is fully relaxed until the Hellmann-Feynman force on each atom is converged to be lower than 0.05eV/Å, and the total energy is converged to 10^{-6} eV during self-consistent calculations. The Brillouin zone is sampled using 2*2*2 Gamma centered k-point mesh. The calculation for defect free UiO-66 is performed in a unit cell with 4 Zn clusters and 24 linkers, and 4 Zn clusters with 8 missing linkers are select to be defected structure. The binding energy is calculated with:

$$E_{binding} = E_{total} - E_{defect} - 16E_{atom}$$

Where E_{defect} is the energy for UiO-66 with 8 missing linkers, E_{atom} is the energy of a single molecular to be adsorbed and E_{total} is the energy after the atoms are adsorbed.

The simulation of electrochemical Zn deposition process is realized by simulation software COMSOL Multiphysics 5.6 with Electrodeposition Tertiary Nernst-Planck. The ions flux was given by the Nernst-Planck equation and the boundary conditions for the electrodes were given by the Butler-Volmer equation. The other boundaries are natural boundaries with no flux. The Zn^{2+} concentration was set to 2 M in the initial state and the resulting ionic concentration was time dependent. The diffusion coefficient of Zn^{2+} in the electrolyte was set to 3.93 e^{-11} m^2 s^{-1}. The current density was set as 20 A m^{-2} The ionic mobilities are defined by Nernst-Einstein equation. The density and molar mass of Zn metal was 7.14 kg m^{-3} and 65.00 g mol^{-1}.

S2 Supplementary Figures and Tables

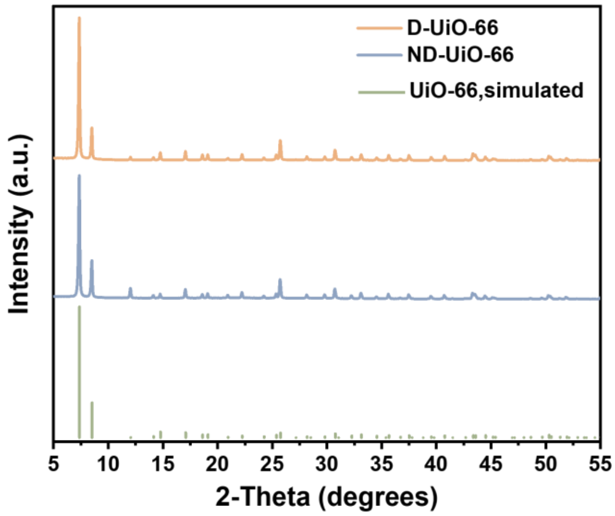

Fig. S1 XRD patterns of D-UiO-66 compared with the ND-UiO-66 and the simulated result

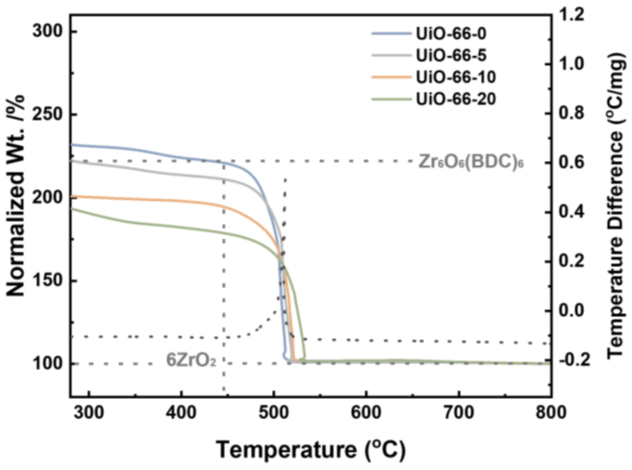

Fig. S2 TGA-DSC curves of UiO-66 synthesized with different HBC content. Solid curves, left axis, TGA trace (relative to ZrO_2). Dotted curves, right axis, DSC trace

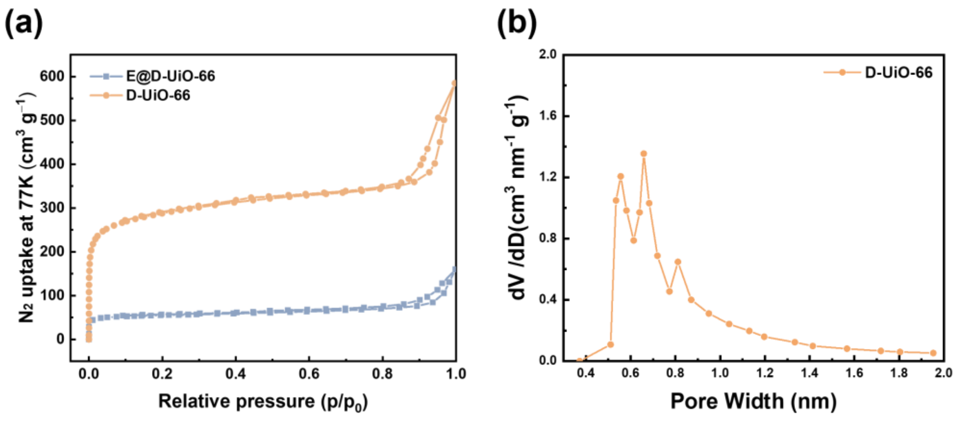

Fig. S3 (**a**) Nitrogen adsorption-desorption isotherms at 77 K of D-UiO-66 and E@D-UiO-66. (**b**) Pore size distributions of D-UiO-66

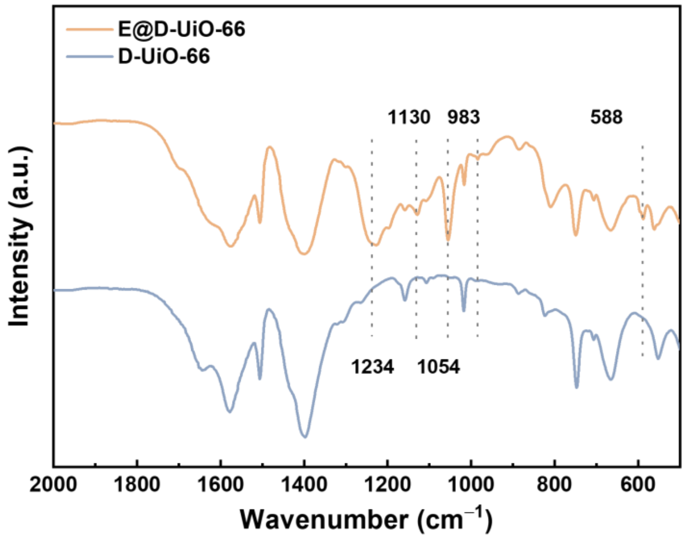

Fig. S4 FTIR spectra of D-UiO-66 and E@D-UiO-66

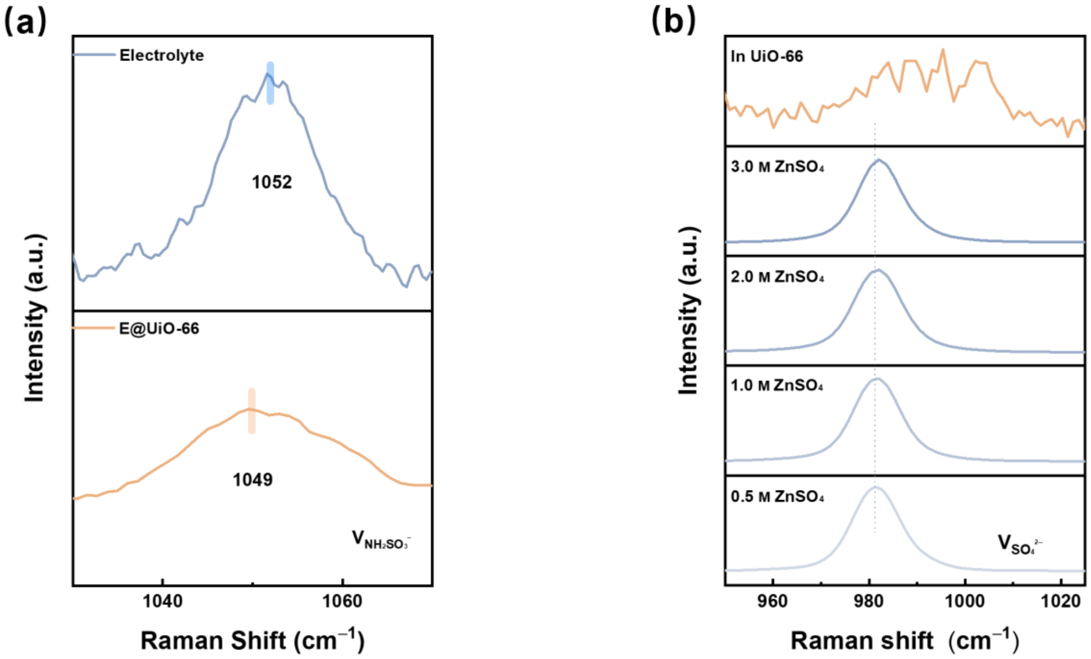

Fig. S5 Enlarged Raman spectra of E@D-UiO-66 in comparison with liquid electrolyte. $\nu_{NH_2SO_3^-}$ is shown in (**a**). Raman spectra of $ZnSO_4$ aqueous solutions with different concentration and 1 M $ZnSO_4$|1 M $Zn(NH_2SO_3)_2$ aqueous solution immersed with D-UiO-66. $\nu_{SO_4^{2-}}$ is shown in (**b**)

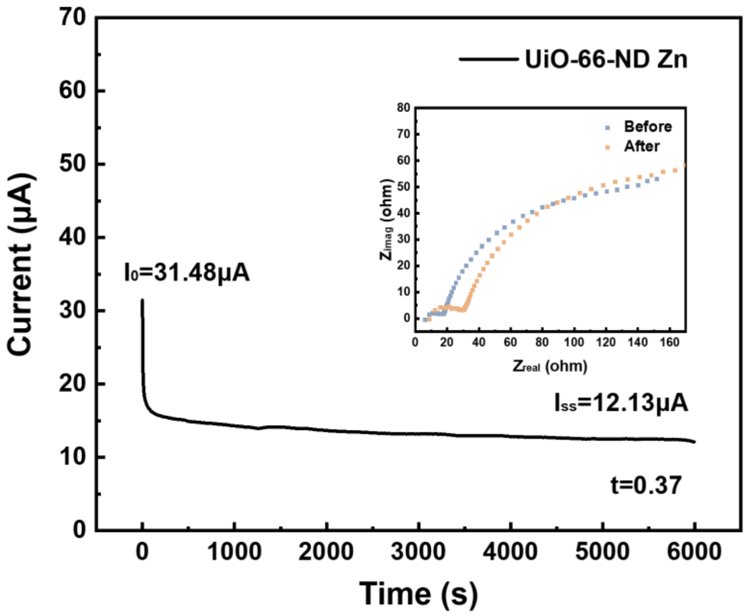

Fig. S6 Current-time profile for ND-UiO-66@Zn||ND-UiO-66@Zn symmetric cell with potentiostatic polarization (∇V = 10 mV). The inset shows AC impedance spectra before and after the following polarization

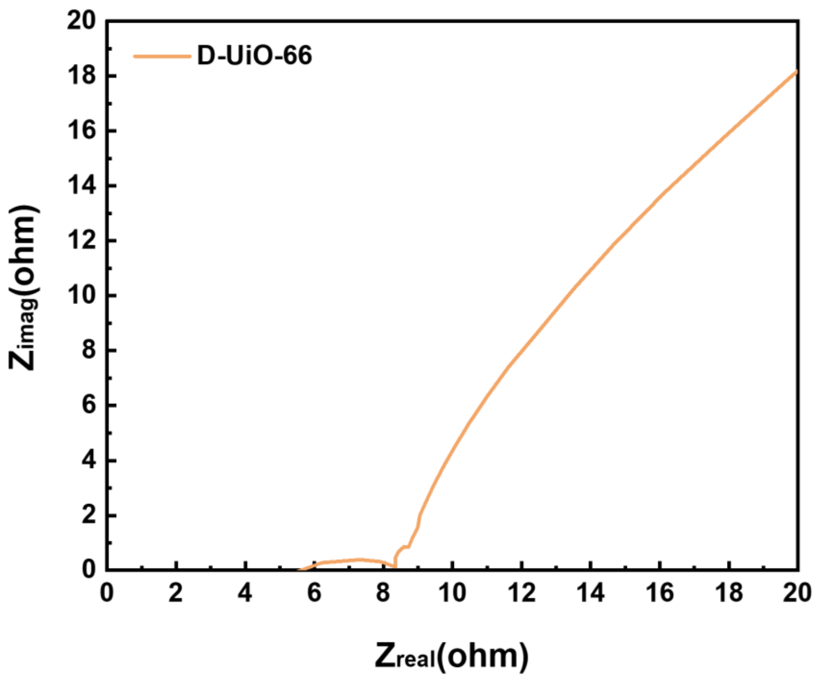

Fig. S7 Nyquist plots over the frequency range of 100 kHz to 1 Hz of D-UiO-66-based quasi-solid interphase

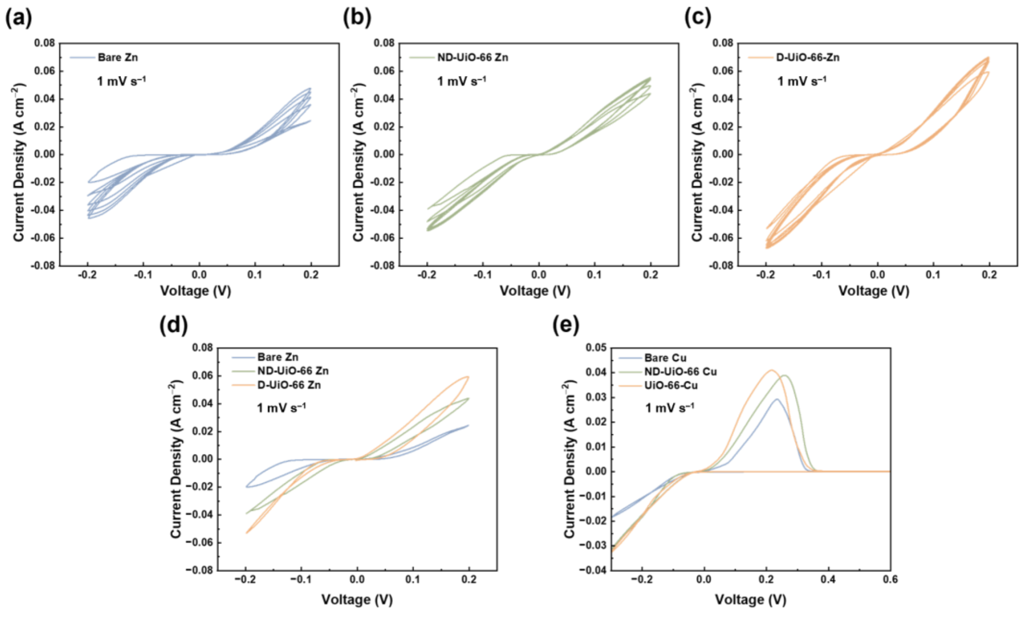

Fig. S8 CV curves scanned at a fixed rate of 1 mV s^{-1} for the first 5 cycles of (**a**) Zn||Zn symmetric cell, (**b**) ND-UiO-66@Zn||ND-UiO-66@Zn symmetric cell and (**c**) D-UiO-66@Zn||D-UiO-66@Zn symmetric cell. (**d**) The comparison of CV curves scanned at a fixed rate of 1 mV s^{-1} for different zinc anodes at the first cycle. (**e**) CV profiles scanned at a fixed rate of 1 mV s^{-1} for Zn nucleation on various Cu substrates

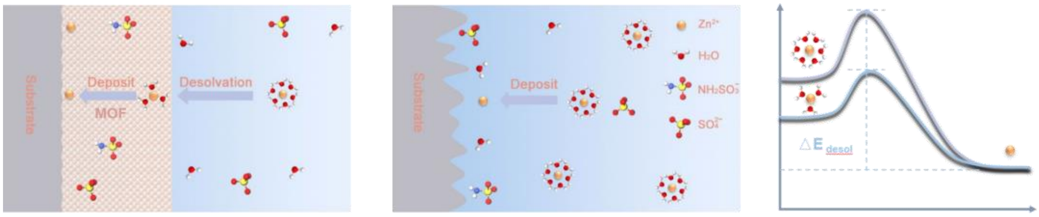

Fig. S9 Schematics of Zn^{2+} desolvation process in MOF-based quasi-solid interphase and liquid electrolyte

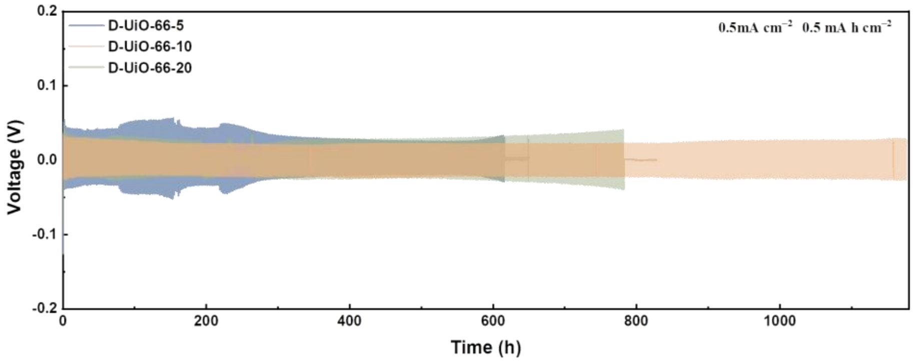

Fig. S10 The electrochemcial performance of Zn symmetric cell tested with different concentration of defects. (Reaction of $ZrCl_4$ and the mixture of H_2BDC and HBC with different ratios in DMF produced as-synthesized UiO-66-*X*, which also represents different concentration of defects.)

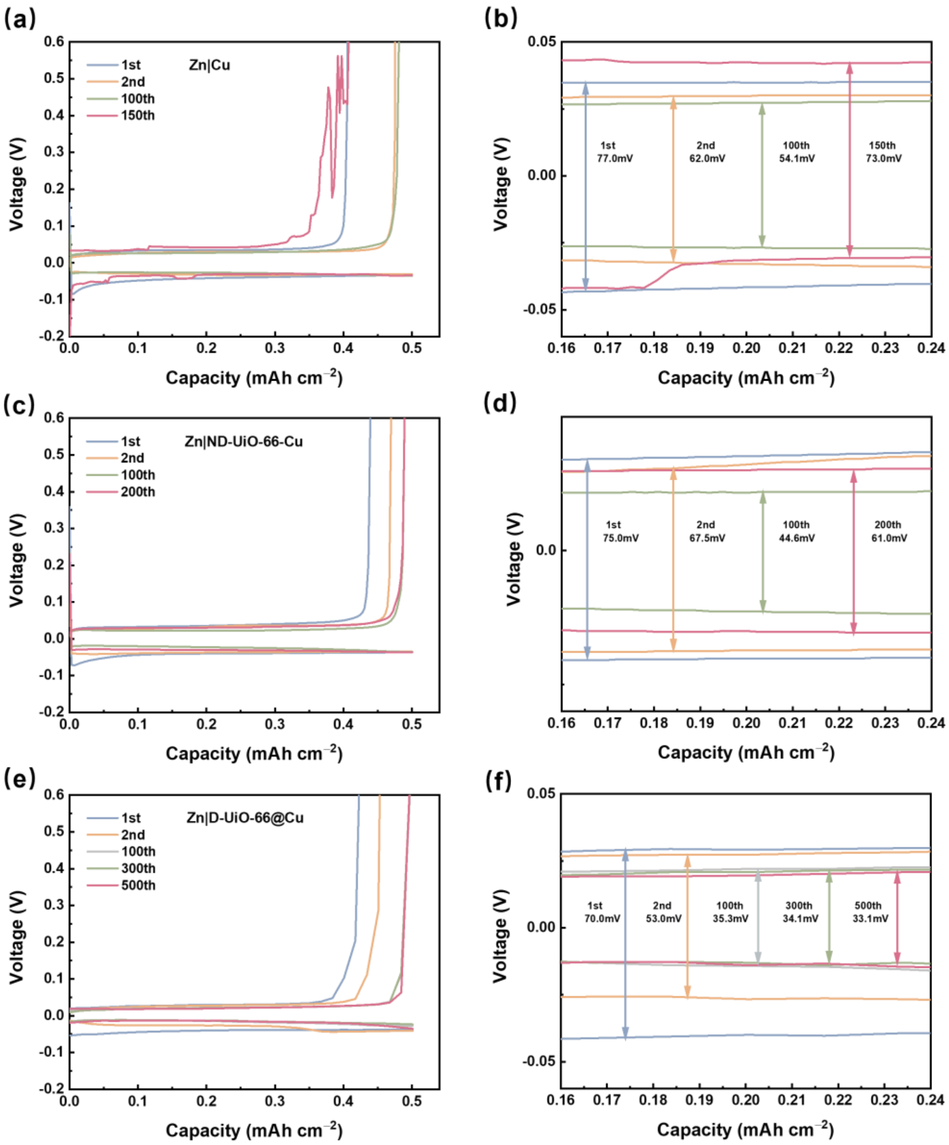

Fig. S11 Voltage profiles of (**a, b**) the Zn||Cu cell, (**c, d**) the Zn||ND-UiO-66@Cu cell, and (**e, f**) Zn||D-UiO-66@Cu cell at selected cycles

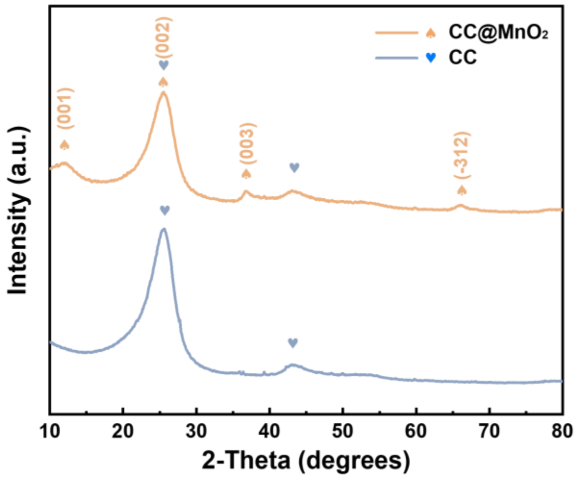

Fig. S12 XRD patterns of CC and CC@MnO_2

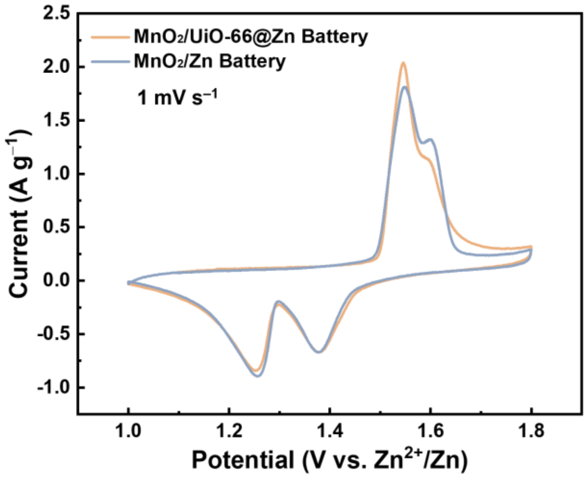

Fig. S13 CV curves of Zn||MnO_2 full cells with and without D-UiO-66 protective layer scanned at a fixed rate of 1 mV s^{-1}

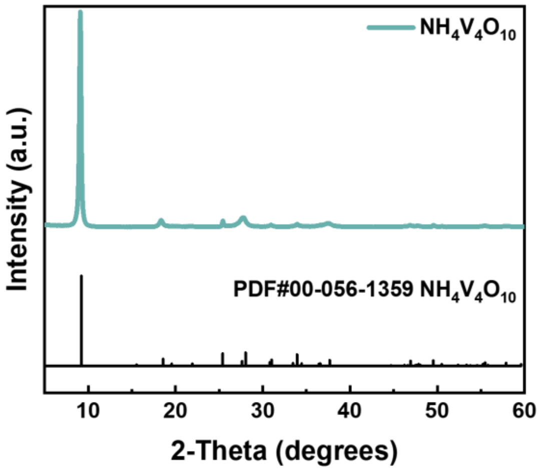

Fig. S14 XRD patterns of $NH_4V_4O_1$

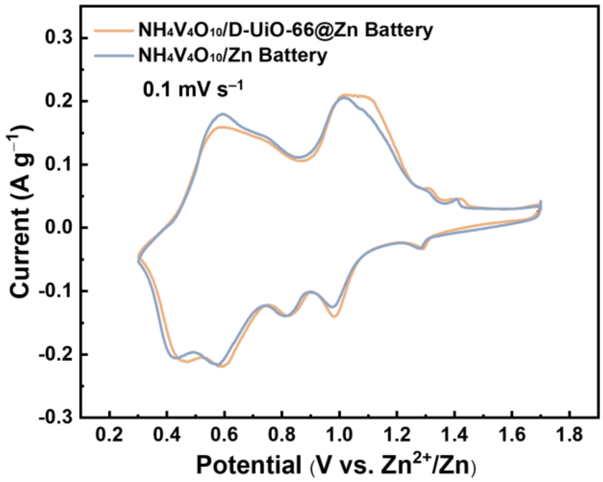

Fig. S15 CV curves of Zn||$NH_4V_4O_{10}$ full cells with and without D-UiO-66 protective layer scanned at a fixed rate of 0.1 mV s^{-1}

Table S1 Estimated the number of missing linkers for UiO-66 (*X*) (*X*= 0, 5, 10, 20) from TGA analyses

Samples	Decomposition Starting Point (% ZrO_2)		Estimated Missing Linkers (per Zr_6 cluster)
	Experimental	Theoretical	
Non-defect UiO-66	220.20	220.20	0.00
Defective UiO-66 (5)	211.05		0.46
Defective UiO-66 (10)	194.58		1.27
Defective UiO-66 (20)	178.72		2.07

Table S2 Vibrational Modes of BDC

**Raman (cm^{-1})**	**description of the mode**
635	benzene ring deformation in terephthalates
1148	in-plane CH bending in terephthalates and benzoates
1437	OCO symmetric stretching in carboxylate in-phase
1453	OCO symmetric stretching in carboxylate in-phase
1618	C=C stretching of aromatic rings

Table S3 The summary of performance applied to Zn in the recent reports using aqueous electrolytes

No.	Electrodes	Electrolyte	*j* (mA cm^{-2})	*C* (mAh cm^{-2})	Life (h)	CC (mAh cm^{-2})	CE (%)	Refs.
1	D-UiO-66@Zn	1 M $ZnSO_4$+1 M $Zn(NH_2SO_3)_2$	10	5	480	2400	99.80	This work
2	ZnS@Zn	1 M $ZnSO_4$	2	2	1100	1100	99.20	[S4]
3	ZnOHF NWs@Zn	2 M $ZnSO_4$	5	1	400	1000	98.80	[S5]
4	PVA@Zn	2 M $Zn(OTf)_2$	10	5	320	1600	99.00	[S6]
5	ZIF-11@Zn	2 M $ZnSO_4$	0.5	0.25	1600	400	99.30	[S7]
6	Zn/CNT anode	2 M $ZnSO_4$	5	0.5	110	275	97.90	[S8]
7	PVB@Zn	1 M $ZnSO_4$	0.5	0.5	2200	550	99.40	[S9]
8	Zn@ZCO	2 M $ZnSO_4$	1	0.25	5000	2500	99.50	[S10]
9	ZCS-Zn	2 M $ZnSO_4$	5	10	800	2000	99.37	[S11]
10	Zn@MCFs	2 M $ZnSO_4$	1	5	1500	1500	99.67	[S12]
11	NA-Zn	3 M $ZnSO_4$	0.25	0.05	2000	250	97.10	[S13]
12	$Ti_3C_2T_x$ MXene@Zn	2 M $ZnSO_4$	1	1	300	150	99.10	[S14]
13	Zn@ZnO-3D	2 M $ZnSO_4$	5	1.25	450	1125	99.55	[S15]
14	Zn@ZnF_2	2 M $ZnSO_4$	10	1	500	2500	99.50	[S16]
15	AEC-Zn	2 M $ZnSO_4$	8.85	8.85	250	1106.25	99.40	[S17]
16	502 glue coated Zn	2 M $ZnSO_4$	2	1	400	400	99.74	[S18]
17	60alucone@Zn	3 M $Zn(OTf)_2$ + 0.1 M $Mn(OTf)_2$	3	1	780	1170	98.60	[S19]
18	PA@Zn	2 M $ZnSO_4$	0.5	0.25	8000	2000	95.12	[S20]
19	Cu–Zn/Zn	3 M $ZnSO_4$	1	0.5	1500	750	91.80	[S21]
20	ZIF-8@Zn	2 M $ZnSO_4$	0.25	0.05	170	21.25	-	[S22]

Supplementary References

[S1] G. Kresse, J. Hafner, Ab initio molecular dynamics for liquid metals. Phys. Rev. B Condens. Matter. **47**, 558-561 (1993). http://doi.org/10.1103/physrevb.47.558

[S2] J.P. Perdew, A. Ruzsinszky, G.I. Csonka, O.A. Vydrov, G.E. Scuseria et al., Restoring the density-gradient expansion for exchange in solids and surfaces. Phys. Rev. Lett. **100**, 136406 (2008). http://doi.org/10.1103/PhysRevLett.100.136406

[S3] S. Grimme, J. Antony, S. Ehrlich, H. Krieg, A consistent and accurate ab initio parametrization of density functional dispersion correction (DFT-D) for the 94 elements H-Pu. J. Chem. Phys. **132**, 154104 (2010). http://doi.org/10.1063/1.3382344

[S4] J. Hao, B. Li, X. Li, X. Zeng, S. Zhang et al., An in-depth study of Zn metal surface chemistry for advanced aqueous Zn-ion batteries. Adv Mater. **32**, e2003021 (2020). http://doi.org/10.1002/adma.202003021

[S5] Z. Pan, Q. Cao, W. Gong, J. Yang, Y. Gao et al., Zincophilic 3D ZnOHF nanowire arrays with ordered and continuous Zn^{2+} Ion modulation layer enable long-term stable Zn metal anodes. Energy Storage Mater. **50**, 435-443 (2022). http://doi.org/10.1016/j.ensm.2022.04.006

[S6] X. Chen, W. Li, S. Hu, N.G. Akhmedov, D. Reed et al., Polyvinyl alcohol coating induced preferred crystallographic orientation in aqueous zinc battery anodes. Nano Energy **98**, 107269 (2022). http://doi.org/10.1016/j.nanoen.2022.107269

[S7] M. He, C. Shu, A. Hu, R. Zheng, M. Li et al., Suppressing dendrite growth and side reactions on Zn metal anode via guiding interfacial anion/cation/H_2O distribution by artificial multi-functional interface layer. Energy Storage Mater. **44**, 452-460 (2022). http://doi.org/10.1016/j.ensm.2021.11.010

[S8] Y. Zeng, X. Zhang, R. Qin, X. Liu, P. Fang et al., Dendrite-free zinc deposition induced by multifunctional CNT frameworks for stable flexible Zn-ion batteries. Adv. Mater. **31**, e1903675 (2019). http://doi.org/10.1002/adma.201903675

[S9] J. Hao, X. Li, S. Zhang, F. Yang, X. Zeng et al., Designing dendrite - free zinc anodes for advanced aqueous zinc batteries. Adv. Funct. Mater. **30**, 2001263 (2020). http://doi.org/10.1002/adfm.202001263

[S10] P. Wang, S. Liang, C. Chen, X. Xie, J. Chen et al., Spontaneous construction of nucleophilic carbonyl-containing interphase toward ultrastable zinc-metal anodes. Adv Mater. **34**, e2202733 (2022). http://doi.org/10.1002/adma.202202733

[S11] Y. Chu, S. Zhang, S. Wu, Z. Hu, G. Cui et al., In situ built interphase with high interface energy and fast kinetics for high performance Zn metal anodes. Energy

Environ. Sci. **14**, 3609-3620 (2021). http://doi.org/10.1039/d1ee00308a

[S12] H. Ying, P. Huang, Z. Zhang, S. Zhang, Q. Han et al., Freestanding and Flexible Interfacial Layer Enables Bottom-Up Zn Deposition Toward Dendrite-Free Aqueous Zn-Ion Batteries. Nano-Micro Lett. **14**, 180 (2022). http://doi.org/10.1007/s40820-022-00921-6

[S13] M. Cui, Y. Xiao, L. Kang, W. Du, Y. Gao et al., Quasi-isolated Au particles as heterogeneous seeds to guide uniform zn deposition for aqueous zinc-ion batteries. ACS Appl. Energy Mater. **2**, 6490-6496 (2019). http://doi.org/10.1021/acsaem.9b01063

[S14] Y. Tian, Y. An, C. Wei, B. Xi, S. Xiong et al., Flexible and free-standing $Ti_3C_2T_x$ MXene@Zn paper for dendrite-free aqueous zinc metal batteries and nonaqueous lithium metal batteries. ACS Nano **13**, 11676-11685 (2019). http://doi.org/10.1021/acsnano.9b05599

[S15] X. Xie, S. Liang, J. Gao, S. Guo, J. Guo et al., Manipulating the ion-transfer kinetics and interface stability for high-performance zinc metal anodes. Energy Environ. Sci. **13**, 503-510 (2020). http://doi.org/10.1039/c9ee03545a

[S16] Y. Yang, C. Liu, Z. Lv, H. Yang, Y. Zhang et al., Synergistic manipulation of Zn^{2+} ion flux and desolvation effect enabled by anodic growth of a 3D ZnF_2 Matrix for long-lifespan and dendrite-free Zn metal anodes. Adv. Mater. **33**, e2007388 (2021). http://doi.org/10.1002/adma.202007388

[S17] R.R. Zhao, Y. Yang, G.X. Liu, R.J. Zhu, J.B. Huang et al., Redirected Zn electrodeposition by an anti-corrosion elastic constraint for highly reversible Zn anodes. Adv. Funct. Mater. **31**, 2001867 (2021). http://doi.org/10.1002/adfm.202001867

[S18] Z. Cao, X. Zhu, D. Xu, P. Dong, M.O.L. Chee et al., Eliminating Zn dendrites by commercial cyanoacrylate adhesive for zinc ion battery. Energy Storage Mater. **36**, 132-138 (2021). http://doi.org/10.1016/j.ensm.2020.12.022

[S19] H. He, J. Liu, Suppressing Zn dendrite growth by molecular layer deposition to enable long-life and deeply rechargeable aqueous Zn anodes. J. Mater.Chem. A. **8**, 22100-22110 (2020). http://doi.org/10.1039/d0ta07232j

[S20] Z. Zhao, J. Zhao, Z. Hu, J. Li, J. Li et al., Long-life and deeply rechargeable aqueous Zn anodes enabled by a multifunctional brightener-inspired interphase. Energy Environ. Sci. **12**, 1938-1949 (2019). http://doi.org/10.1039/c9ee00596j

[S21] Z. Cai, Y. Ou, J. Wang, R. Xiao, L. Fu et al., Chemically resistant Cu–Zn/Zn composite anode for long cycling aqueous batteries. Energy Storage Mater. **27**, 205-211 (2020). http://doi.org/10.1016/j.ensm.2020.01.032

[S22] X. Pu, B. Jiang, X. Wang, W. Liu, L. Dong et al., High-performance aqueous zinc-ion batteries realized by MOF materials. Nano-Micro Lett. **12**, 152 (2020). http://doi.org/10.1007/s40820-020-00487-1
